# Human-derived fecal microbiota transplantation alleviates social deficits of the BTBR mouse model of autism through a potential mechanism involving vitamin B_6_ metabolism

**DOI:** 10.1128/msystems.00257-24

**Published:** 2024-05-23

**Authors:** Lifeng Zheng, Yinming Jiao, Haolin Zhong, Yan Tan, Yiming Yin, Yanhong Liu, Ding Liu, Manli Wu, Guoyun Wang, Jinqun Huang, Ping Wang, Meirong Qin, Mingbang Wang, Yang Xiao, Tiying Lv, Yangzi Luo, Han Hu, Sheng-Tao Hou, Ling Kui

**Affiliations:** 1 Brain Research Centre and Department of Neuroscience, Southern University of Science and Technology, Shenzhen, China; 2 Xbiome Co. Ltd., Shenzhen, China; 3 Shenzhen Qianhai Shekou Free Trade Zone Hospital, Shenzhen, China; 4 Shenzhen Institute for Drug Control, Shenzhen, China; 5 Microbiome Therapy Center, South China Hospital, Medical School, Shenzhen University, Shenzhen, China; 6 Shanghai Key Laboratory of Birth Defects, Division of Neonatology, Children’s Hospital of Fudan University, National Center for Children’s Health, Shanghai, China; 7 Department of Hematology, Shenzhen Qianhai Shekou Free Trade Zone Hospital, Shenzhen, China; 8 Guangzhou University of Chinese Medicine, Guangzhou, China; 9 Faculty of Biology, Medicine and Health, The University of Manchester, Manchester, United Kingdom; Chan Zuckerberg Biohub, Stanford, California, USA

**Keywords:** fecal microbiota transplantation (FMT), autism spectrum disorder (ASD), BTBR, social deficit, vitamin B_6 _metabolism, fatty acid metabolism, *Bacteroides*, pyridoxal 5′-phosphate (PLP)

## Abstract

Autism spectrum disorder (ASD) is a heterogeneous neurodevelopmental condition characterized by social communication deficiencies and stereotypic behaviors influenced by hereditary and/or environmental risk factors. There are currently no approved medications for treating the core symptoms of ASD. Human fecal microbiota transplantation (FMT) has emerged as a potential intervention to improve autistic symptoms, but the underlying mechanisms are not fully understood. In this study, we evaluated the effects of human-derived FMT on behavioral and multi-omics profiles of the BTBR mice, an established model for ASD. FMT effectively alleviated the social deficits in the BTBR mice and normalized their distinct plasma metabolic profile, notably reducing the elevated long-chain acylcarnitines. Integrative analysis linked these phenotypic changes to specific *Bacteroides* species and vitamin B_6_ metabolism. Indeed, vitamin B_6_ supplementation improved the social behaviors in BTBR mice. Collectively, these findings shed new light on the interplay between FMT and vitamin B_6_ metabolism and revealed a potential mechanism underlying the therapeutic role of FMT in ASD.

**IMPORTANCE:**

Accumulating evidence supports the beneficial effects of human fecal microbiota transplantation (FMT) on symptoms associated with autism spectrum disorder (ASD). However, the precise mechanism by which FMT induces a shift in the microbiota and leads to symptom improvement remains incompletely understood. This study integrated data from colon-content metagenomics, colon-content metabolomics, and plasma metabolomics to investigate the effects of FMT treatment on the BTBR mouse model for ASD. The analysis linked the amelioration of social deficits following FMT treatment to the restoration of mitochondrial function and the modulation of vitamin B_6_ metabolism. Bacterial species and compounds with beneficial roles in vitamin B_6_ metabolism and mitochondrial function may further contribute to improving FMT products and designing novel therapies for ASD treatment.

## INTRODUCTION

Autism spectrum disorder (ASD) constitutes a group of neurodevelopmental disorders characterized by impaired social communication and restricted, repetitive behaviors (1). The prevalence of ASD in children in the United States has been steadily increasing, rising from 1 in 150 in 2000 to 1 in 36 in 2020, with males affected four times more frequently than females (2). ASD was assumed to be caused by environmental factors (3, 4) and genetic abnormalities (5–7). The U.S. Food and Drug Administration (FDA) has approved only risperidone and aripiprazole for treating irritability in ASD patients (8). There is an urgent need for innovative medications targeting the core symptoms of ASD, especially social deficits.

Recent research has bolstered the concept of a functional gut-brain axis (GBA), establishing a connection between gut microbiota and brain activity in autism (9, 10) as well as other neuropsychiatric disorders (11, 12). Microbiome compositions differed between individuals with ASD and those with typically developing (TD) brains (13–16), evolving as ASD individuals aged (17), though inconsistencies exist between studies (18). The beneficial impact of microbiome therapeutics on ASD-related behaviors has been explored in animal models (19, 20) and ASD children (21). Microbes may either trigger or alleviate ASD symptoms by interacting with intestinal epithelium (22), immune and neural systems (23), regulating neurotransmitters (16, 24), and modulating amino acid metabolism (25). Notably, fecal microbiota transplantation (FMT) has been applied to ASD children in a few clinical studies (26–29), achieving promising results in both gastrointestinal (GI) symptoms and behaviors, with an efficacy rate of 64.6% in some studies (30). Due to the promising preliminary results of FMT in ASD (26, 27), the FDA has fast-tracked an FMT therapy for children with ASD in 2019 (31). Despite the clinical progress, the mechanisms and efficacy of FMT are likely donor- and recipient-dependent. Using genetically identical mice with human-derived FMT allows us to focus only on the donor part in the context of ASD. Understanding the mechanisms and key components of FMT could aid in refining FMT treatment and products for ASD.

To investigate the efficacy of FMT in alleviating ASD symptoms, microbiota from a healthy adult male donor was administered to BTBR mice. FMT is commonly used in ASD mouse model studies to establish a causal relationship between the microbiome and host phenotype (32–35). In this study, FMT successfully rescued social deficits while leaving stereotypic behavior unaffected in mice. Post-FMT treatment, significant changes occurred in both metabolome and metagenome profiles, with many alterations associated with disturbed fatty acid metabolism, indicative of mitochondrial dysfunction in the host. The multi-omics analysis also hinted at the potential roles of vitamin B_6_ metabolism and associated *Bacteroides* species in the host-microbe interaction. In a subsequent proof-of-concept study, the effectiveness of vitamin B_6_ in mitigating social behavior deficits in BTBR mice was confirmed. These findings contribute to our understanding of the mechanisms behind FMT treatment for ASD and propose vitamin B_6_ metabolism as a potential therapeutic target in ASD management.

## RESULTS

### FMT ameliorated the impaired social behaviors of the BTBR mice

**Fig 1:**
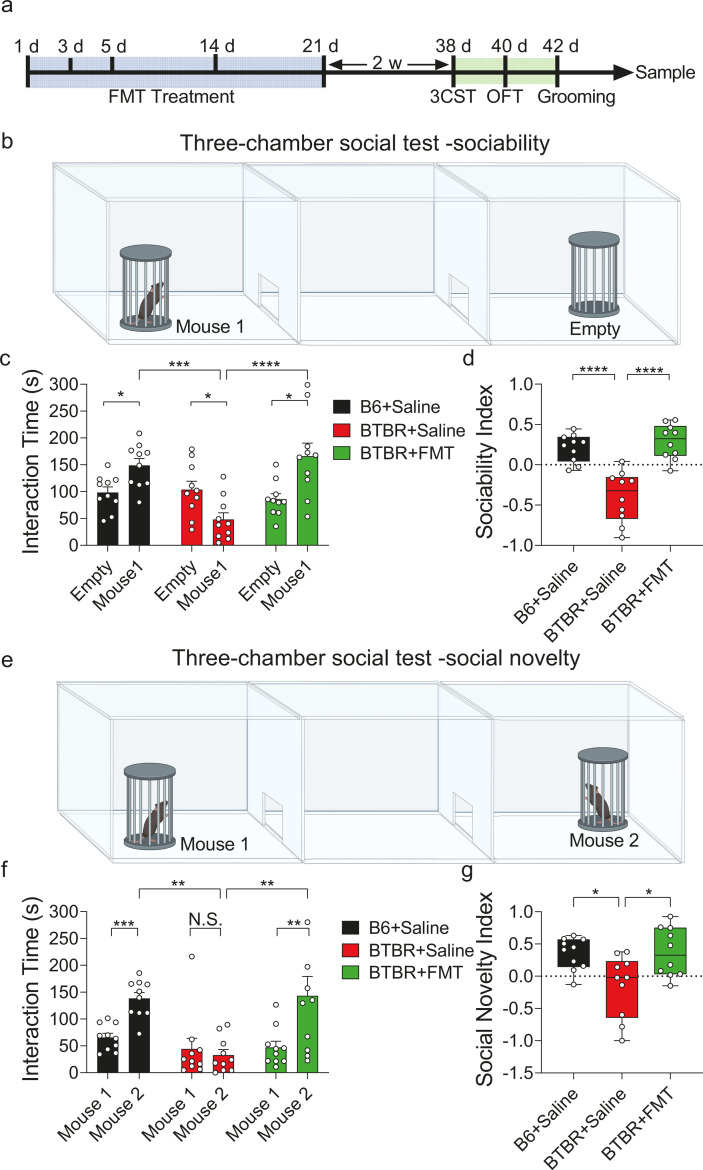
Human FMT rescued the social deficit of the BTBR mice. (**a**) The timeline of fecal microbiota transplantation in mice and scheme of study design. (**b**) Schematic of the sociability task in a three-chamber social behavior test. (**c**) Interaction time of experiment subject with a stranger mouse or empty cage in sociability task. (**d**) Sociability index of the BTBR+FMT group, the BTBR+Saline group, and the B6+Saline group (*n* = 10 mice in each group). (**e**) Schematic of the social novelty task in a three-chamber social behavior test. (**f**) Interaction time of experiment subject with a familiar mouse or unfamiliar one in sociability task. (**g**) Social novelty index of the BTBR+FMT group, the BTBR+Saline group, and the B6+Saline group (*n* = 10 mice in each group). Data represent the mean ± SEM. Error bars indicate SEM. *P < 0.05, **P < 0.01, ***P < 0.001, ****P < 0.0001. Two-way ANOVA with Tukey’s post hoc test was used in panels c and f, and one-way ANOVA with Dunnett’s post hoc test was used in panels d and g. The graphical element in panels b and e were created using BioRender (https://biorender.com).

FMT of the human microbiome was applied to the BTBR model to investigate the mechanism underlying FMT treatment for ASD. The selection of BTBR mice as an ASD model is based on the resemblance of their abnormal behaviors to the core deficits observed in ASD (36, 37). Previous research has indicated that BTBR mice have a unique gut microbial community (38, 39), and cohousing with C57BL/6J (B6) mice can ameliorate their social deficits (40). In our study, the BTBR mice were administered either FMT from a male adult donor (BTBR+FMT group) or saline (BTBR+Saline group) five times within 3 weeks (Fig. 1a). As a control for ASD-like behaviors, B6 mice were given saline (B6+Saline) according to the same protocol. Two weeks after FMT administration, the three-chamber social test (3CST), open field test (OFT), and grooming test were performed to evaluate the efficacy of the treatment on the social, anxiety-like, and stereotypic behaviors of the BTBR and B6 mice (41, 42), respectively.

To validate the autism model, we compared the behavior test results of the BTBR+Saline and B6+Saline groups. In the sociability test, the duration of interaction for the experimental mouse was assessed, either with a stranger mouse (Mouse 1) or an empty wired cup (Fig. 1b). As expected, B6+Saline mice exhibited normal social behavior, as evidenced by their preference for interacting with the unfamiliar mouse. In contrast, the BTBR+Saline mice did not prefer mouse 1, indicating impaired sociability (two-way ANOVA *F*_(2,54)_ = 11.07, *P* < 0.0001, Tukey’s post hoc test: *P* = 0.1180, Fig. 1c). Meanwhile, the sociability index of the BTBR+Saline mice was significantly lower than that of the B6+Saline group (one-way ANOVA *F*_(2, 27)_ = 23.98, *P* < 0.0001, Dunnett’s post hoc test: *P* < 0.0001, Fig. 1d). In addition, the social novelty analysis (Fig. 1e) demonstrated that mice in the B6+Saline group spent significantly more time interacting with the novel mouse (Mouse 2) than in the BTBR+Saline group (two-way ANOVA *F*_(2,54)_ = 4.536, *P* = 0.0151, Tukey’s post hoc test: *P* = 0.0028, Fig. 1f; one-way ANOVA *F*_(2, 27)_ =1.496, *P* = 0.0075, Dunnett’s post hoc test: *P* = 0.0134, Fig. 1g). Compared to the B6 mice, mice in the BTBR+Saline group spent less time in the center zone and more time grooming themselves during the open field test (one-way ANOVA *F*_(2, 27)_ = 1.063, *P* = 0.0413, Dunnett’s post hoc test: *P* = 0.0231, Fig. S1a; one-way ANOVA *F*_(2, 27)_ = 6.500, *P* < 0.0001, Dunnett’s post hoc test: *P* < 0.0001, Fig. S1b), which indicated their anxiety-like and stereotypic behaviors, respectively.

The study proceeded to assess the impact of FMT by comparing the behavioral improvements between the BTBR+FMT group and the BTBR+Saline group. Intriguingly, treatment with human FMT rescued the sociability of BTBR mice, as measured by the time spent with mouse 1 (two-way ANOVA *F*_(2,54)_ = 11.07, *P* < 0.0001, Tukey’s post hoc test: *P* < 0.0001, Fig. 1c), and elevated the sociability index in the BTBR+FMT group to the level of the control group (B6 mice) (one-way ANOVA *F*_(2, 27)_ = 23.98, *P* < 0.0001, Dunnett’s post hoc test: *P* < 0.0001, Fig. 1d). Meanwhile, mice in the BTBR+FMT group showed a usual preference for social novelty, as they spent more time interacting with Mouse 2 (novel one) than Mouse 1 (familiar one) (two-way ANOVA F_(2,54)_ = 4.536, *P* = 0.0151, Tukey’s post hoc test: *P* = 0.0015, Fig. 1f). The social novelty index of the BTBR+FMT group also increased significantly compared to the BTBR+Saline group one-way ANOVA *F*_(2, 27)_ = 1.496, *P* = 0.0075, Dunnett’s post hoc test: *P* = 0.0100, Fig. 1g).

Besides, mice in the BTBR+FMT group still displayed abnormal anxiety after FMT treatment, in which mice spent more time in the periphery than the center in the open field test (one-way ANOVA *F*_(2, 27)_ = 1.063, *P* = 0.0413, Dunnett’s post hoc test: *P* = 0.2987, Fig. S1a). Meanwhile, FMT failed to alleviate the stereotypic behaviors of BTBR mice, as seen by the unchanged accumulated duration of self-grooming with FMT treatment (one-way ANOVA *F*_(2, 27)_ = 6.500, *P* < 0.0001, Dunnett’s post hoc test: *P* = 0.9975, Fig. S1b). This finding is unsurprising since social activity and stereotypic behaviors may undergo disparate mechanisms (43, 44), and the FMT efficacy is also donor-dependent (45).

In summary, treatment with human gut microbiota selectively reversed ASD-like social deficit symptoms in BTBR mice.

### The FMT-treated BTBR mice harbored specific bacterial species potentially associated with enhanced social behaviors

**Fig 2:**
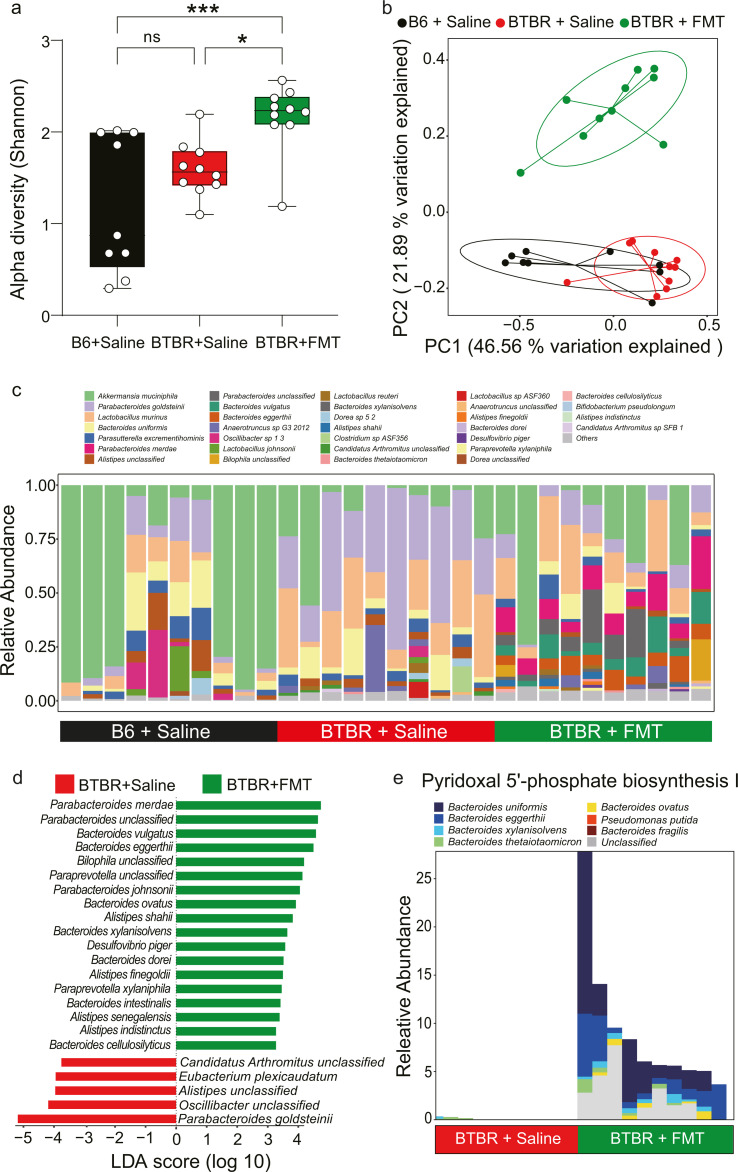
Human FMT altered the colon-content microbiome in the BTBR mice. (**a**) Alpha diversity (Shannon index) between mice in the B6+Saline, BTBT+Saline, and BTBR+FMT groups were compared (*n* = 10 per group). Horizontal lines represent the third quartile, median, and first quartile, respectively. The whiskers above and below the box show the maximum and minimum. (**b**) PCoA analysis of the microbiome profiles of the three groups B6+Saline, BTBR+Saline, and BTBR+FMT (*R*^2^ = 0.279, *P* = 0.006, PERMANOVA analysis). (**c**) Comparison of the genus-level taxonomic composition of the microbiome between the B6+Saline, BTBR+Saline, and BTBR+FMT groups. (**d**) LEfSe analysis showing differentially abundant bacterial species between the BTBR+FMT and the BTBR+Saline groups (LDA score cutoff set as 2.0). (**e**) Bacteria species contributing to the pathway of pyridoxal 5′-phosphate Biosynthesis I in the BTBR+Saline and the BTBR+FMT. The stacked bar indicates the bacteria-stratified abundance of the pathway within a single sample.

Investigating the impact of FMT on the colon-content microbiome profile in BTBR mice, we observed no significant difference in α-diversity between the BTBR+Saline and B6+Saline groups (Fig. 2a). In contrast, BTBR mice treated with FMT showed significantly higher α-diversity values compared to both the B6 and BTBR mice treated with saline (Fig. 2a). β-diversity analysis based on Bray-Curtis distance showed that samples from the BTBR+FMT group were shifted away from the other two groups (PERMANOVA test, *R*^2^ = 0.441, *P* = 0.001, Fig. 2b) and the BTBR+Saline samples separated from the B6+Saline group (PERMANOVA test, *R*^2^ = 0.301, *P* = 0.002, Fig. S2a). This suggested that the human-derived FMT caused substantial changes in the microbiome of the BTBR mice, which was also distinct from the normal microbiome profile of the B6 mice. Comparison between the BTBR+Saline and B6+Saline groups identified 13 differential species using LEfSe (46) (LDA > 2, *P* < 0.05) (Fig. S2b). *Akkermansia muciniphila* and *Olsenella* unclassified were significantly increased in the B6 group. We then compared the taxonomic compositions between the BTBR+FMT and BTBR+Saline groups. Multiple species from *Parabacteroides*, *Bacteroides*, *Alistipes, Bilophila*, and so on, were present in the BTBR+FMT samples and the donor sample but depleted in the BTBR+Saline samples (Fig. 2c; Table S1), suggesting the donor origin of these species in the BTBR+FMT group. We performed differential analysis between the BTBR+FMT and BTBR+Saline groups using LefSe (46) (LDA > 2, *P* < 0.05) and identified 23 species (Fig. 2d). Most species enriched in the BTBR+FMT group belonged to the genera *Bacteroides*, *Parabacteroides,* and *Alistipes*, although species from genera *Bilophila*, *Paraprevotella*, and *Desulfovibrio* were also increased in the group (Fig. 2d). The results were consistent with previous animal and clinical studies: *B. ovatus* and *P. merdae* were absent in offspring ASD mice (32); *Parabacteroides*, *Alistipes*, and *Bilophila* were decreased in ASD individuals (14); *Paraprevotella* and *Bacteroides* were found to decrease in ASD children (47); and *D. piger* was found to increase in ASD children after receiving FMT treatment (48). The enriched species have been recognized for their beneficial effects in ASD, including correction of gut permeability (19), and modulation of neurotransmitters such as γ-aminobutyric acid (GABA) (49, 50), glutamate (50), and tryptophan (51). The species decreased in the BTBR+FMT group included *Eubacterium plexicaudatum*, *Parabacteroides goldsteinii*, and unclassified species from *Candidatus* Arthromitus, *Alistipes*, and *Oscillibacter* (Fig. 2d). *C.* Arthromitus was enriched in ASD mice and significantly decreased after FMT treatment (52). *P. goldsteinii* has been reported to be beneficial to ASD-like behaviors in MIA offspring (53), though it was more abundant in our study in the BTBR+Saline group (Fig. 2c and d). The exact roles of these species in ASD require further investigation.

Functional changes introduced to the microbiome by the FMT treatment were further examined using HUMAnN 2 (54). From the pathway analysis results, we identified 24 differentially regulated pathways (|log_2_(FC)| > 1, *P* < 0.05) (Fig. S3a). HUMAnN 2 (54) also provided a pathway abundance table stratified by taxa to illustrate the contribution of specific taxa in the pathways. Notably, the pathway “pyridoxal 5'-phosphate biosynthesis I” (pathway #11 in Fig. S3a) was enriched in the BTBR+FMT group with multiple *Bacteroides* species (*B. uniformis*, *B. eggerthii*, *B. xylanisolvens*, *B. thetaiotaomicron*, and *B. ovatus*) involved (Fig. 2e). Pyridoxal 5′-phosphate (PLP) is the active form of vitamin B_6_, which acts as a co-factor involved in the biosynthesis of multiple neurotransmitters, including serotonin, dopamine, norepinephrine, epinephrine, and GABA (55). Gut microbiota can mediate vitamin B_6_ homeostasis and regulate autism-like behavior in a mice model (56), but the generalizability of the model and the underlying mechanisms in ASD are not yet fully understood.

We correlated the differential species with behavior indices across the BTBR mice samples to investigate the connection between ASD-like behaviors and microbiome members (Fig. S4a). The results showed that the sociability index was positively correlated with most species enriched in the BTBR+FMT group and negatively correlated with those depleted in the BTBR+FMT group, suggesting strong variation of both microbiome abundance and sociability post-FMT treatment. In addition, the social novelty index was negatively associated with *Alistipes* unclassified, and positively correlated with *Alistipes senegalensis*. The anxiety behavior index (time in the center of open field test) was positively associated with *Paraprevotella* unclassified, and the stereotypic behavior index (grooming duration time) was negatively associated with *Ca.* Arthromitus.

### Metabolomics of colon contents linked FMT treatment to lowered toxins, increased protective compounds, and altered neurotransmitter regulation

**Fig 3:**
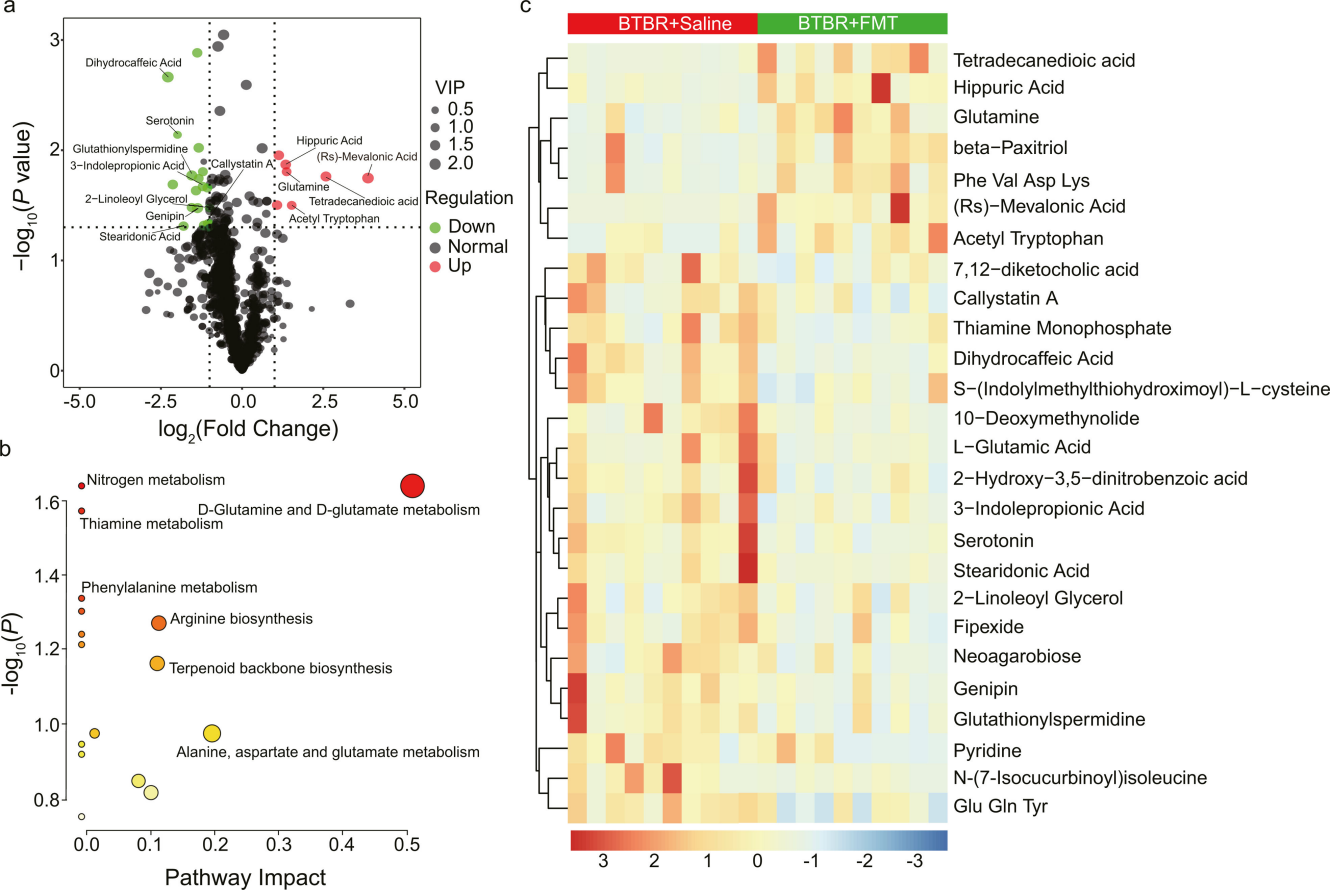
The effect of FMT treatment on the colon-content metabolome in the BTBR mice. (**a**) The volcano plot showing the variations of colon-content metabolites between the BTBR+FMT group and the BTBR+Saline group. The dots represented in green (downregulated) and red (upregulated) are differentially regulated metabolites with |log_2_(fold change) | > 1, a *P* value < 0.05, and VIP value > 1.0. VIP value represents the importance projection value of the metabolite obtained from the PLS-DA model. (**b**) Bubble plot showing the metabolic pathways altered by FMT treatment in BTBR mice. The bubble size corresponds to the impact of the pathway, while the color gradient represents the levels of significance, transitioning from the highest (red) to the lowest (white). (**c**) Heatmap depicting the differential metabolites between the BTBR+FMT and BTBR+Saline groups. Metabolite abundances were normalized to a constant sum and scaled by Pareto scaling. The color gradient, ranging from red (high abundance) to blue (low abundance), represents metabolite levels on a log_2_ scale.

Principle component analysis (PCA) revealed that the samples from the BTBR+FMT and BTBR+Saline groups could not be distinguished based on their colon contents metabolite profiles; while samples from the B6+Saline group clustered separately (Fig. S2c). Subsequent statistical and pathway analyses using MetaboAnalyst (57) identified 26 differential metabolites (|log_2_(FC)| > 1, *P* < 0.05, VIP > 1) between the BTBR+FMT and the BTBR+Saline groups (Fig. 3a). Pathway enrichment analysis with MetaboAnalyst identified 16 pathways, where pathway “*D*-glutamine and *D-*glutamate metabolism” had the highest impact value (0.5) and a small *P* value (*P* = 0.024) (Fig. 3b).

Due to the complex origins of the metabolites in the colon contents, they were expected to have a multifaceted influence on ASD. In fact, some of the differential metabolites have been reported as biomarkers of ASD, including hippuric acid (58), glutamine/glutamate (59), and serotonin (60); some are related to neurotransmitter metabolism, including serotonin (61), 5-methoxy tryptamine (5-MT, serotonin agonist, structurally related to melatonin), 3-indole propionic acid (3-IPA; tryptophan metabolite) (62), and glutamine/glutamate (63). Besides, some metabolites are associated with beneficial biological functions associated to ASD, such as cholesterol homeostasis (MVA) (64), mitochondria function (tetradecanedioic acid) (65, 66), anti-inflammation (stearidonic acid) (67), neuroprotection (*N-*acetyl-tryptophan) (68), dihydrocaffeic acid (69), human cannabinoid type 1 (CB1) receptor-mediated memory, and neurohormone regulation (70, 71) (2-linoleoyl glycerol, or 2-LG, partial agonist of CB1 receptor). We also noticed a decrease in toxicants in the BTBR+FMT group, including genipin (72), callystatin A (73), and glutathionylspermidine (74). This finding is consistent with the theory that environmental toxicants contribute to the impairment of mitochondrial function in ASD patients who miss detoxification capability (75).

Comparison between the BTBR+Saline and B6+Saline groups revealed 293 differential metabolites (|log_2_(FC)| > 1, *P* < 0.05, VIP > 1) (Fig. S2e). Pathway analysis between the two groups illustrated “Alanine, aspartate and glutamate metabolism,” “Linoleic acid metabolism,” “Riboflavin metabolism,” “Arginine and proline metabolism,” and “Vitamin B_6_ metabolism” as top enriched pathways (raw *P* < 0.05, Impact > 0.5) (Fig. S2g), which did not overlap with the pathways altered by FMT treatment in the BTBR mice (Fig. 3b).

In short, the colon-content metabolome changes in the BTBR+FMT group suggested the beneficial roles of FMT in reducing toxicants and increasing protective metabolites for mitochondria and neurological health. FMT may also contribute to the modulation of tryptophan-serotonin metabolism and glutamine/glutamate balance in the gut.

### Plasma metabolomics analysis identified altered metabolites related to GABAergic signaling and mitochondrial function

Compared to the colon-content metabolome, the plasma metabolome was closely related to the physiological status of the host. As anticipated, PCA analysis of the plasma metabolome revealed a distinct separation between samples from the B6+Saline group, the BTBR+Saline group, and the BTBR+FMT group (Fig. S2d). The BTBR+FMT group was closer to the BTBR+Saline group than to the B6+Saline group (Fig. S2d), suggesting that mouse strain is a key factor shaping the profile of the plasma metabolome.

**Fig 4:**
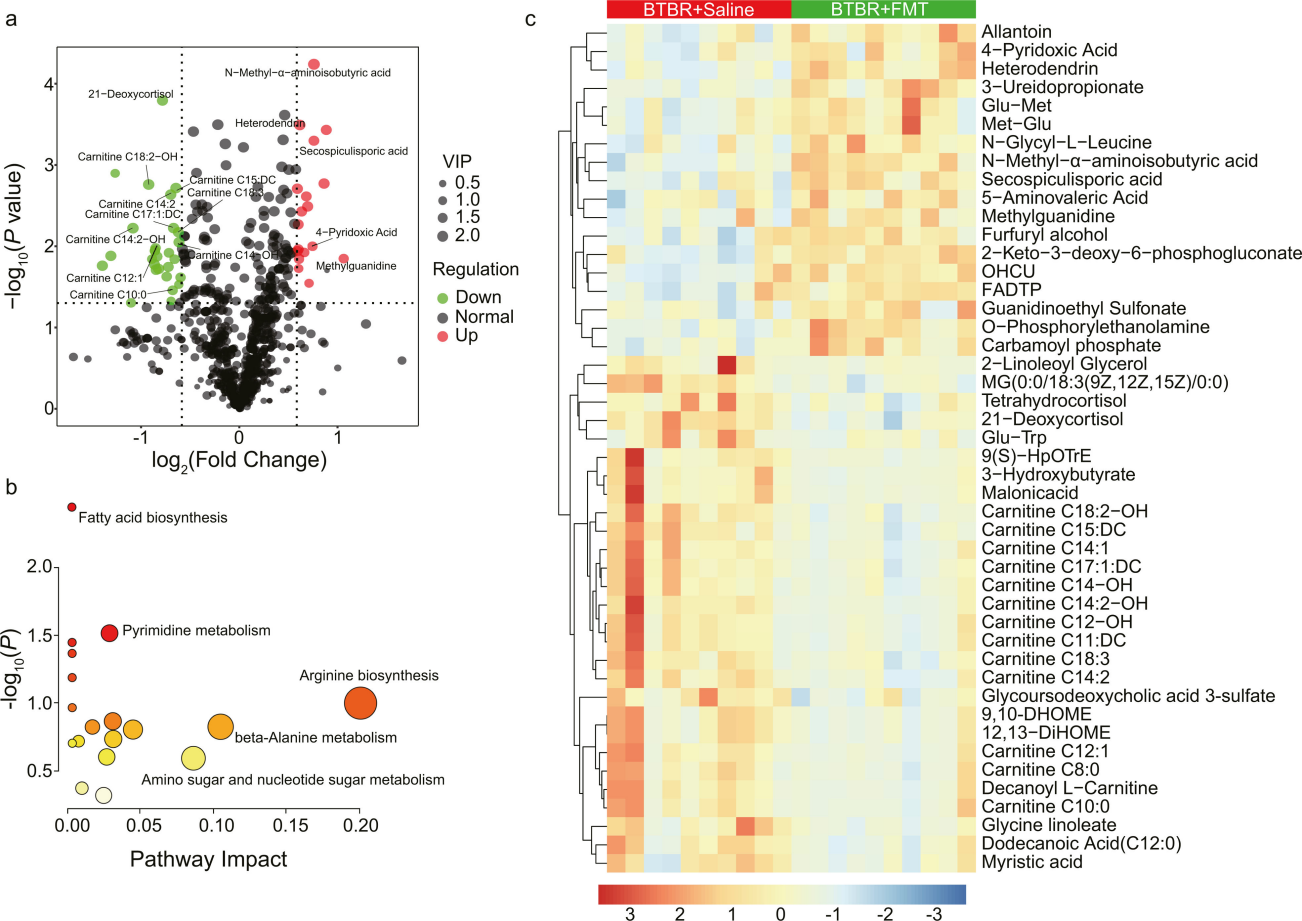
The effect of FMT treatment on the plasma metabolome in the BTBR mice. (**a**) The volcano plot shows the variations of plasma metabolites between the BTBR+FMT group and the BTBR+Saline group. The dots represented in green (downregulated) and red (upregulated) are differentially regulated metabolites with |log_2_(fold change) | > 1, a *P* value < 0.05, and VIP value > 1.0. VIP value represents the importance projection value of the metabolite obtained from the PLS-DA model. (**b**) Bubble plot showing the alteration of pathways by FMT treatment in the BTBR mice. The bubble size corresponds to the impact of the pathway, while the color gradient represents the levels of significance, transitioning from the highest (red) to the lowest (white). (**c**) Heatmap depicting the differential metabolites between the BTBR+FMT group and the BTBR+Saline group mice. Metabolite abundances were normalized to constant sum and scaled by Pareto scaling. The color gradient, ranging from red (high abundance) to blue (low abundance), represents metabolite levels on a log_2_ scale.

Metabolomics analysis of the data identified five decreased metabolites and one increased metabolite in the BTBR+FMT group (VIP > 1, *P* < 0.05, |log_2_(FC)| > 1). The decreased metabolites included malonic acid (MA), 3-hydroxybutyrate, 2-LG, carnitine C14:2-OH, and MG(0:0/18:3(9Z,12Z,15Z)/0:0). Notably, 2-LG was also identified as a differential metabolite in the colon-content metabolome. MG (0:0/18:3(9Z,12Z,15Z)/0:0), or 2-monolinolenin, is a structural analog of 2-LG and may share similar functions. Methylguanidine (MG), the only metabolite that increased significantly, is an oxidative stress biomarker that reflects the changes in arginine metabolism. It can inhibit nitric oxide (NO) formation (76), critical in regulating cell survival, differentiation, and proliferation of neurons in autism and other neurological disorders (77, 78). Perturbation of MG level has been observed in the urine of ASD children (79, 80). To expand the hypothesis space, we relaxed the cutoff of fold change to 1.5 (>1.5 or <2/3) and identified 18 increased metabolites and 28 decreased metabolites in the BTBR+FMT group (Fig. 4a). The following pathway analysis reported 18 pathways in total, including pyrimidine metabolism, arginine biosynthesis, β-alanine metabolism, amino sugar and nucleotide sugar metabolism, and so on (Fig. 4b). Amino acid and urea cycle disorders have been associated with ASD pathogenesis (81, 82). However, these pathways exhibited either significant raw *P* values (*P* > 0.05, or equivalently, −log_10_(*P*) < 1.3) or shallow impact values (approaching 0), as shown in Fig. 4b. This diminishes the reliability of functional interpretations derived from these pathways.

Further examination of the differential metabolites revealed the enrichment of multiple long-chain acylcarnitine derivatives in the BTBR+Saline group. These include carnitine C10:0, carnitine C11: DC, carnitine C12:1, carnitine 14-OH, carnitine 14:1, carnitine 14:2, carnitine 14:2-OH, carnitine 15: DC, carnitine C17:1: DC, carnitine C18:2-OH, and carnitine C18:3 (Fig. 4c). This pattern suggests impaired fatty acid β-oxidation and has been proposed as a biomarker for ASD children with mitochondrial dysfunction (83). Note that the pathway analysis did not reveal any pathways related to long-chain acylcarnitine derivatives, owing to the missing ID hits of these compounds (Table S2). Besides, MA and 3-hydroxybutyrate have also been reported to be involved in mitochondrial function (84, 85). An increase in a subset of metabolites related to GABAergic signaling was observed in the BTBR+FMT group, including 5-amino valeric acid (5-AV; a methylene homolog of GABA), guanidinoethyl sulfonate (GES; an analog of taurine), and *O*-phosphorylethanolamine (PEA; an analog of GABA). Interestingly, 5-AV and taurine are GABA_A_ agonists and were demonstrated by Sharon et al. to improve social and repetitive behaviors in BTBR mice (32). 5-AV was also found to be decreased in urine samples from a group of individuals with ASD (86). PEA has been proposed as a biomarker for ASD (87). Additionally, an increase in 4-pyridoxic acid was observed in the BTBR+FMT group, which is the catabolic product of vitamin B_6_, reflecting the status of vitamin B_6_ and renal function in the host (88).

Comparison between the BTBR+Saline group and the B6+Saline group identified 69 differential metabolites (|log_2_(FC)| > 1, *P* < 0.05, VIP > 1) (Fig. S2f). Pathway analysis between the BTBR+Saline and B6+Saline groups highlighted “Histidine metabolism,” “beta-Alanine metabolism,” “Arginine biosynthesis,” “Alanine, aspartate and glutamate metabolism,” “Linoleic acid metabolism,” and “Glycine, serine and threonine metabolism” as top enriched pathways (raw *P* < 0.05, Impact > 0.5) (Fig. S2h).

### Integration of multi-omics results: tracing the path from the FMT treatment to social behavior improvement

We seek to better understand FMT’s paths to alleviate the social behavior deficits in the BTBR mouse model. We performed correlation analysis between each omics data set and the behavioral indices (Fig. S4a through c) and between the omics data sets themselves (Fig. S5). Notably, a significant portion of the differential taxa and metabolites were correlated with the sociability index.

**Fig 5:**
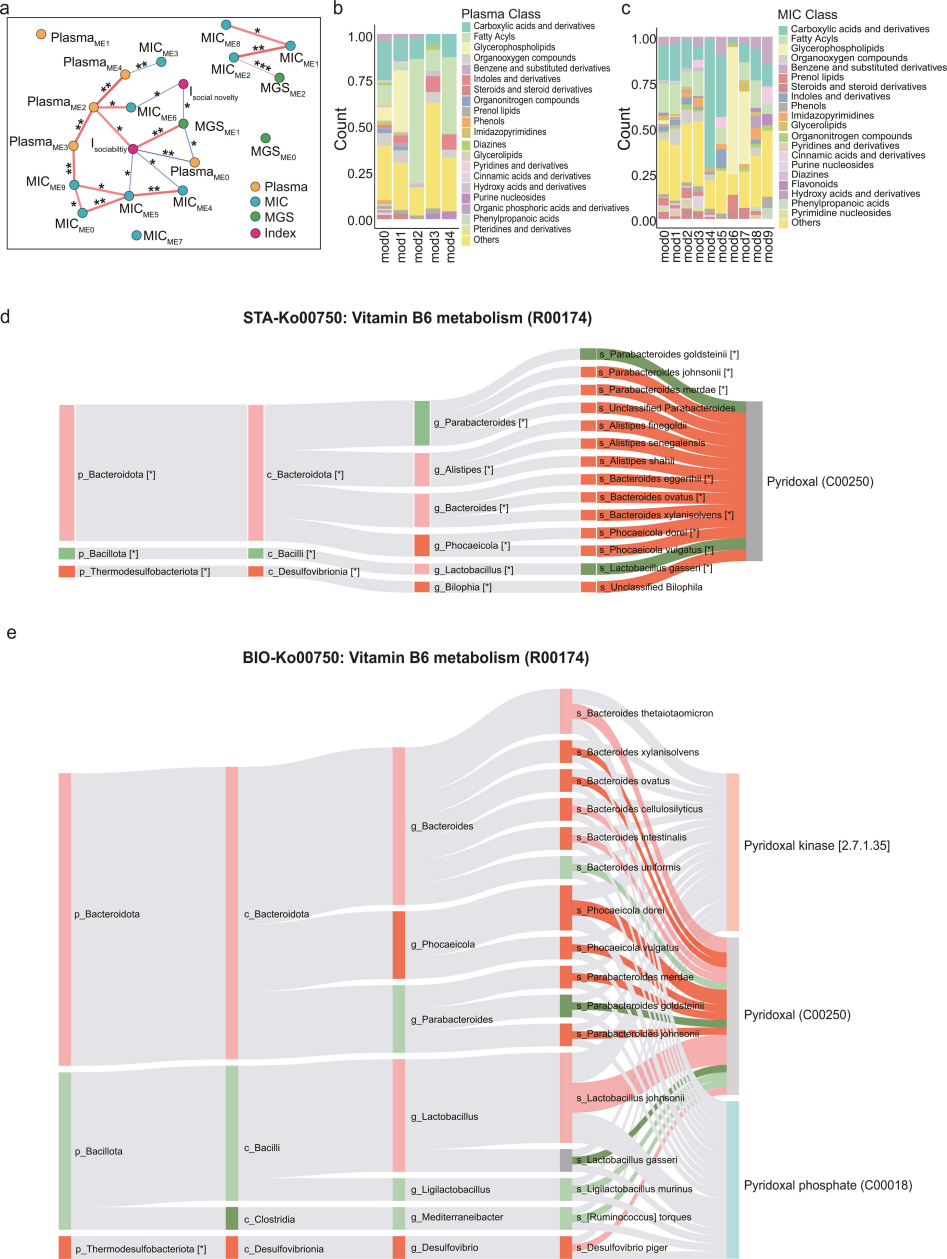
Integration and analysis of multi-omics data reveal key contributors to social behavior change in BTBR mice. (**a**) Correlation network between sociability and social novelty indices and WGCNA modules derived from each omics data set. Orange dots represent plasma metabolites modules, blue dots represent the colon-content metabolites modules, and green dots represent the metagenome modules. Modules were represented by the module eigen genes. Pearson correlation was used for the correlation coefficients. Asterisks indicate the significant correlations between modules. *P < 0.05, **P < 0.01, ***P < 0.001. (**b**) The distributions of metabolite classes within WGCNA modules derived from the plasma metabolomics data set. (**c**) The distribution of metabolite classes within WGCNA modules derived from the colon-content metabolomics data set. (**d**) The STA-Sankey network for R00174 metabolic reaction in vitamin B_6_ metabolism. Spearman’s rank correlation was used for the correlation coefficients. Asterisks indicate taxa with statistical significance. Node color: red indicates upregulation, green indicates downregulation. (**e**) The BIO-Sankey network for R00174 metabolic reaction in vitamin B_6_ metabolism. Band color: red indicates positive, and green indicates negative correlations with metabolites. Deep red/green on bands denote statistically significant correlations (*P*  <  0.05).

To explore the major contributors to the phenotype change in each omics data set, we conducted weighted gene co-expression analysis (WGCNA) to extract modules from each data set across the BTBR mice samples (see “Integration of multi-omics data” below). The WGCNA analysis extracted three modules from the metagenomics data set (MGS), ten from the colon-content metabolomics (MIC), and five from the plasma metabolomics (PL) (Table S3). We also measured the correlation between these modules and the sociability index (Fig. 5a). Significant associations were found between the sociability index and several modules: Module 1 (MGS), Module 0 (PL), Module 2 (PL), Module 4 (MIC), and Module 5 (MIC). Additionally, the social novelty index correlated with Module 1 (MGS) and Module 6 (MIC) (Fig. 5a). Module 1 (MGS), the only module from the metagenomics data that correlated with the sociability index, predominantly included most *Alistipes* species and two *Bacteroides* species (*B. ovatus* and *B. xylanisolvens*) (Table S3), suggesting their significant roles in improving social behavior in the BTBR mouse model. In plasma metabolomics, Module 2 (PL) was dominated by long-chain acylcarnitine derivatives and other compounds related to fatty acyls, as shown in Fig. 5b, aligning with the results from the previous differential analysis (Fig. 4c). Module 4 (MIC) and Module 5 (MIC) comprised a variety of oligopeptides (classified as “Carboxylic acids and derivatives,” as shown in Fig. 5c; Table S3). Module 6 (MIC) included glycerophospholipids such as lysophosphatidylcholine (LPCs) and phosphatidylethanolamine (PPEs) (Fig. 5c; Table S3). Most compounds were not observed in the differential analysis in the colon-content metabolomics (Fig. 3c).

In parallel, we performed MetOrigin analysis (89) to extract the relationships between bacteria species and metabolites (see “Integration of multi-omics data” below). MetOrigin is designed to trace metabolites to different origins, such as food, host, or co-metabolism, and provides statistical and biological association information for the connections it identifies. Paired analysis was performed between metagenomics and plasma metabolomics (M-P), as well as between metagenomics and colon-content metabolomics (M-C), separately. In the M-C analysis, MetOrigin identified four pathways as originating from “Co-metabolism,” one pathway from “Microbiota,” and one pathway from the “Host” (Fig. S6**)** . Similarly, the M-P analysis reported six pathways as “Co-metabolism” origin, one pathway as “Microbiota” and one as “Host” (Fig. S7). Notably, the plasma compound pyridoxal was traced to multiple *Bacteroides* (*Phocaeicola*) spp., *Parabacteroides* spp., and *L. gasseri*, a connection supported by both statistical and biological relationships (Fig. 5d and e). Pyridoxal is a natural form of vitamin B_6_. It can be converted to PLP by pyridoxal kinase. This finding aligns with the results of stratified pathway analysis in metagenomics previously, which indicated that multiple *Bacteroides* species capable of PLP synthesis (a part of vitamin B_6_ metabolism) were enriched in the BTBR+FMT group (Fig. 2e). Given that vitamin B_6_ is essential for the metabolism of long-chain unsaturated fatty acid (90, 91), a process crucial for normal mitochondria function, we suspected that vitamin B_6_ metabolism may bridge the gap between the altered *Bacteroides* species brought by FMT and subsequent metabolic and phenotypic changes in the BTBR mice.

### Vitamin B6 alleviates social behavior deficits similar to FMT in the BTBR mouse model

**Fig 6:**
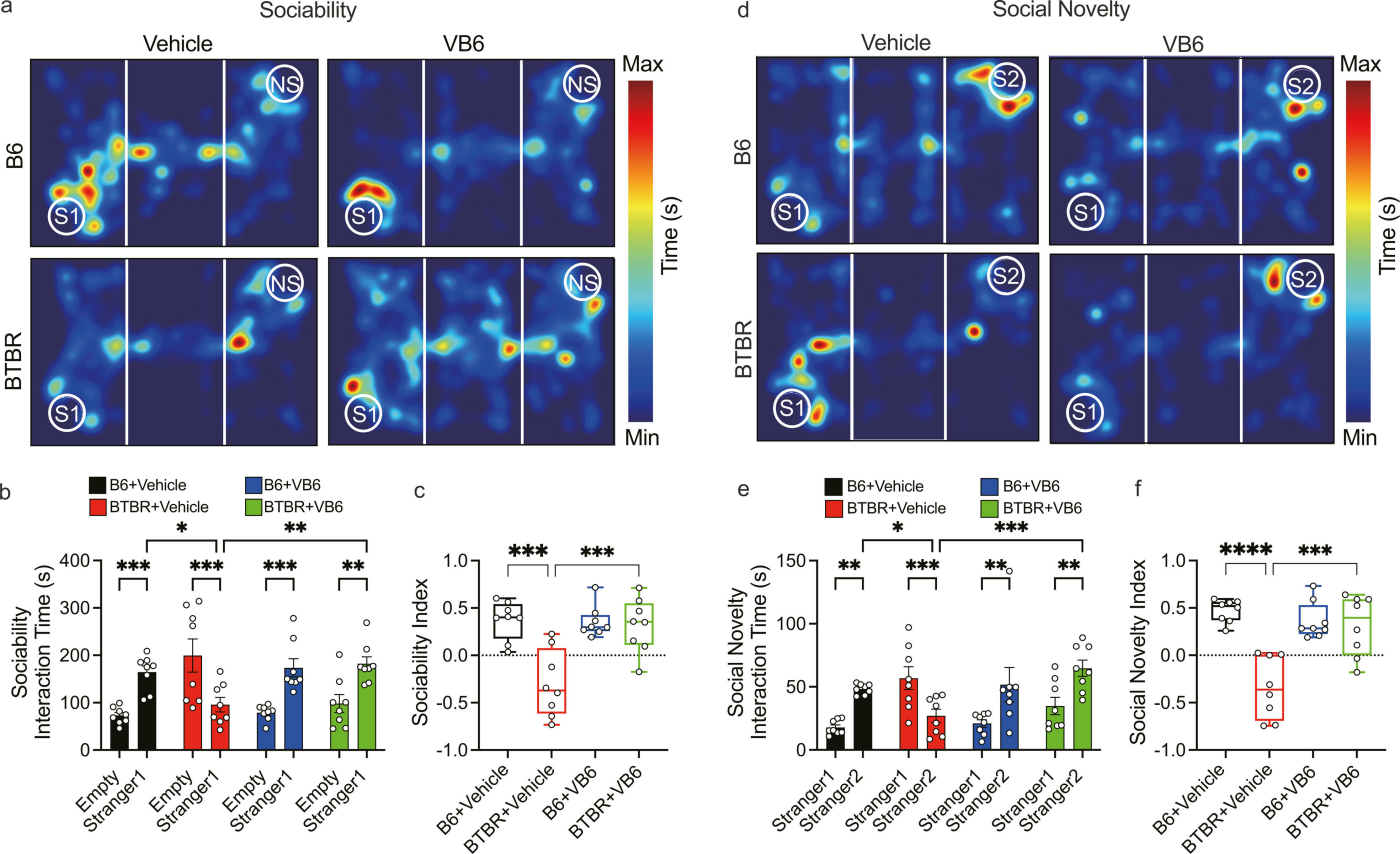
Vitamin B_6_ alleviated the social behavior of the BTBR mice. (**a**) Representative heatmaps of the three-chamber sociability test performed in the B6 and BTBR mice. (**b**) Interaction time of experiment subject with a stranger mouse or empty cage in sociability task. (**c**) Sociability index of B6 and BTBR mice (*n* = 8 mice in each group). (**d**) Representative heatmaps of the three-chamber social novelty test performed in B6 and BTBR mice. (**e**) Interaction time of experiment subject with a stranger mouse or a familiar mouse in social novelty task. (**F**) Social novelty index of B6 and BTRB mice (*n* = 8 mice in each group). Data represent the mean ± SEM. Error bars indicate SEM. *P < 0.05, **P < 0.01, ***P < 0.001, ****P < 0.0001. The test used in (b, c, e, f): two-way ANOVA with Tukey’s post hoc test.

As a proof of concept, the effectiveness of vitamin B_6_ was tested in the mouse model (Fig. 6; Fig. S1). Intriguingly, treatment with vitamin B_6_ remarkably affected the sociability (Fig. 6a) and social novelty (Fig. 6d) of the BTBR mice. Indeed, the sociability performance of vitamin B_6_-treated BTBR mice resembled that of the B6+Vehicle group and the B6+VB_6_ group (Fig. 6b and c). Compared to the BTBR+Vehicle group, the BTBR+VB_6_ group exhibited a reversal in the social novelty deficit, showing increased interaction time with the novel mouse (Fig. 6e and f). However, vitamin B_6_ did not alleviate anxiety and repetitive behavior in the BTBR mice (Fig. S1c and d), which is consistent with the efficacy performance of the BTBR+FMT group (Fig. S1a and b).

## DISCUSSION

Although significant advances have been made in both animal (32–34, 92, 93) and clinical studies (26–28), the efficacy of current FMT treatments for ASD heavily depends on donor selection and remains a black box. In our study, plasma metabolomics analysis revealed a distinct acylcarnitine profile in the BTBR+Saline group, suggesting impaired fatty acid β-oxidation and mitochondrial dysfunction (94, 95) in the ASD model (96, 97). FMT can normalize the impaired fatty acid metabolism (Fig. 3C), with changes in acylcarnitine levels negatively correlating with improvements in the social behavior index (Fig. S4c). Mitochondrial function is known to be crucial for the social behavior of BTBR mice (98) and is a prominent issue in ASD children (99). The concept of mitochondrial dysfunction in ASD pathogenesis has been extensively discussed in both animal (96, 97) and human studies (83, 100, 101). Future research will delve deeper into mitochondrial functionality in the context of ASD and its connection to social behavior.

Multiple components in FMT may contribute to the normalization of fatty acid metabolism or mitochondrial function. Our colon-content metabolomics analysis indicated that FMT increased metabolites beneficial for mitochondrial function and neurological health while reducing toxicants. The relationship between these metabolites and mitochondrial function appears complex and multifaceted, necessitating extensive research to elucidate these interactions fully. Nonetheless, our results highlighted a potential connection via vitamin B_6_ metabolism: pathway analysis of the metagenomics data identified an enrichment of *Bacteroides* species (*B. uniformis*, *B. eggerthii*, *B. xylanisolvens*, *B. thetaiotaomicron*, and *B. ovatus*) with genes involved in PLP synthesis in the BTBR+FMT group (Fig. 2e). Differential analysis of the plasma metabolomics identified an increase of 4-pyridoxic acid, the end product of vitamin B_6_ metabolism, in the BTBR+FMT group (Fig. 4c). Further, integrative multi-omics analysis using MetOrigin (89) also associated the *Bacteroides* species with plasma levels of pyridoxal (Fig. 5d and e), suggesting their roles in modulating vitamin B_6_ metabolism. The level of 4-pyridoxic acid was positively correlated with *B. ovatus* and *B. eggerthii*, while pyridoxal was positively correlated with *B. ovatus*, *B. eggerthii* and *B. xylanisolvens* (Fig. S4d). Note that *B. ovatus* and *B. xylanisolvens* were also present in Module 1 (WGS). In a proof-of-concept study, we validated the effectiveness of vitamin B_6_ in mitigating social behaviors in the BTBR mouse model. Although a previous animal study reported the role of gut microbiota in alleviating ASD symptoms via vitamin B_6_ homeostasis (56), our study further suggested the connection between gut microbiota-regulated vitamin B_6_ metabolism and impaired fatty acid metabolism (suggesting mitochondria dysfunction) in the context of ASD mice model. Future research should investigate the interplay between these pathways and their collective impact on improving social behavior in ASD.

Additional beneficial elements in FMT may also contribute to the improvement of sociability. In the BTBR+FMT group, we noted alterations in GABAergic signaling in the plasma metabolomics data. Observing altered compounds, such as 5-AV and taurine analog, aligns with previous reports that GABA_A_ receptor agonists like 5-AV and taurine promoted GABAergic signaling and aided in autism symptom alleviation (32). Concurrently, metagenomics analysis showed an increase in *Bacteroides*, *Parabacteroides*, and *Alistipes* species. *B. ovatus* can module the levels of some intestinal neurotransmitters, for example, increasing the intestinal GABA and decreasing tryptophan and glutamine (49). *B vulgatus and B. dorei* can decrease the production of gut microbial lipopolysaccharide (102). *P. merdae* can protect against seizure by reducing systemic GABA and elevating hippocampal GABA and glutamate levels (50). Many *Alistipes* species modulate tryptophan metabolism by hydrolyzing tryptophan into indole derivatives (51) and participate in preserving gut barrier integrity (103). In our study, *A. senegalensis* was negatively associated with undesirable metabolites such as fipexide, genipin, and glutathionylspermidine (Fig. S5a). Our study did not cover the enriched species' multiple effects, necessitating a detailed examination of specific species or even individual strains to understand their roles and impacts fully.

Our study has several limitations. First, we did not include an FMT-treated B6 group as a control. Comparing the FMT-treated BTBR group with an FMT-treated B6 group would aid in identifying bacterial candidates that uniquely persist in the BTBR+FMT group and may be associated with behavior improvements. Additionally, our study only inferred the roles of specific bacterial species in alleviating social deficits in the BTBR mouse model. Validating the effects experimentally using strains isolated from the same donor and replicating the experiments with different animal models is crucial to confirm these findings. Furthermore, we did not eliminate the impact of bacteria originating from the BTBR mice with antibiotic treatment. Although many species present in the BTBR mice after FMT treatment were exclusive to the human donor samples (Table S1), there remains the possibility that the donor’s microbiome may interact with the native microbiome of the mice, resulting in a novel community with unique functions.

Future work should focus on refining the effective components of FMT and elucidating their connections with ASD. It’s important to recognize that many ASD cases do not involve mitochondrial dysfunction or a disorder of vitamin B_6_ metabolism. Therefore, it would be valuable to compare responses to FMT treatment between ASD children with and without mitochondrial dysfunction. Moreover, stereotypic behaviors in ASD are mediated through different mechanisms (104). Investigating donors or treatments that primarily target stereotypic behaviors could lead to the development of combinatory therapies addressing both core symptoms of ASD. Such research could significantly enhance our understanding of the disease and improve treatment strategies.

## MATERIALS AND METHODS

### Animals

Three-month-old male C57Bl/6 mice and BTBRT^+^Itpr3^tf^/J mice were purchased from the Jackson Laboratory (USA). Mice were maintained in a pathogen-free SPFII animal facility in a condition-controlled room (23 ± 1°C, 50 ± 10% humidity). A 12 h light/dark cycle was automatically imposed. Mice were housed in groups of four to five per individually ventilated cages and given access to food and water *ad libitum* unless otherwise stated in the methods below. Experimenters were blinded to the treatment of animals and sample processing throughout the subsequent experimentation and analyses.

### Fecal microbiota transplantation

Healthy donors were screened and recruited by Shenzhen Xbiome Biotech Co. Ltd. Human fecal samples from a healthy adult male were collected at the Shenzhen Xbiome Biotech Co. Ltd according to the study protocol approved by. Informed consent was obtained from the subject. Fecal samples were suspended in an equal volume (wt/vol) of phosphate-buffered saline (PBS) containing 20% glycerol in PBS, snap-frozen in liquid nitrogen, and stored at −80°C until use. The frozen stocks were thawed, suspended in saline, filtered through a 100-µm cell strainer, and orally inoculated into BTBR mice (approximately 3.64 × 10^10^ CFU/mL, 200 µL per mouse). Control groups were administered saline.

### Mouse behavioral tests

Transfer rodents to the procedure room (for acclimation) 1 h before testing. The apparatus was carefully cleaned by wiping it with 75% ethanol after each experiment to remove the olfactory distraction. The person performing the test was blind to the animal groups. All mice were familiarized with the researchers at least one week in advance.

### Open field test

Mouse spontaneous activity in the open field was tested using a clear plastic cube box (*L* × *W* × *H*: 41 × 41 × 38 cm^3^) under a camera. The center region of the box field was defined as a 20 cm × 20 cm virtual area. Each mouse was given 10 min to move freely in the box. All traveled distances and the distance trace center were recorded and analyzed using Noldus EthoVision XT (V15, Noldus Information Technology, Leesburg, VA, USA). The rearing numbers, representing the number of times mice lifted the forepaws to explore upwards, were recorded by the experimenter.

### Self-grooming test

The mice were placed in a clean observation box (*L* × *W* × *H*: 20 cm × 20 cm × 50 cm) with bedding on the bottom under a camera. With low light conditions (about 40 Lux), the behavior of mice was recorded for 10 min after pre-adaptation to the observation box. The experimenter remained quiet and away from the test mice during the test. The duration of the grooming of mice was recorded and analyzed using captured videos.

### Three-chamber social test

Mice were first habituated for 10 min in an empty (40 cm × 60 cm × 22 cm) Plexiglass arena divided into three interconnected chambers (left, center, and right). Sociability was evaluated during a second 10-min period in which the subject could interact either with an empty wire cup (Empty) or a wire cup containing a genotype, age, sex, and treatment-matched stranger conspecific (Mouse 1). The interaction time was determined by measuring the time the subject mouse spent sniffing or climbing upon either the empty cup or the cup containing the stranger mouse. The position of the empty cup/stranger mouse in the left or right chamber during the sociability period was counterbalanced between trials to avoid bias. Preference for social novelty was assayed in a third 10-min period by introducing a second stranger mouse (Mouse 2) into the previously empty wire cup. The time spent interacting with the empty cup or Mouse 1 or Mouse 2 was recorded and measured using Noldus EthoVision XT (V15, Noldus Information Technology, Leesburg, VA, USA). Sociability index = (*T*_mouse 1_− *T*_empty_)/(*T*_mouse 1_ + *T*_empty_), social novelty index = (*T*_mouse 2_ − *T*_mouse 1_)/(*T*_mouse 2_ + *T*_mouse 1_).

### Behavior test statistics

Data were presented as mean ± SEM. Statistical analyses performed include one- or two-way ANOVA with Bonferroni post hoc analysis to correct for multiple comparisons unless otherwise indicated. *P*, *t*, and *F* values were presented in the figure legends. *P* < 0.05 was considered statistically significant: *P < 0.05, **P < 0.01, ***P < 0.001, ****P < 0.0001. Prism 9 (version 9; GraphPad, CA, USA) software was used to perform statistical analyses and generate graphical data representations.

### Fecal DNA extraction, library preparation, and sequencing

Colon-content samples were collected from BTBR mice, as previously described. Briefly, DNA was extracted using the QIAamp PowerFecal DNA Kit (catalog no. 51804, Qiagen), followed by library construction and sequencing on an Illumina NovoSeq 6000 platform (Novo Gene). Stool samples were collected from BTBR mice, as previously described. Briefly, DNA was extracted using the QIAamp PowerFecal DNA Kit (catalog no. 51804, Qiagen), followed by library construction and sequencing on an Illumina NovoSeq 6000 platform (Novo Gene).

### Metagenomics analysis

Quality control of raw reads and host decontamination were performed with KneadData (version 0.6.1, reference genome hg19 for human donor data decontamination, and mm10 for mice data decontamination). Taxonomic profiling and microbial pathway abundance analysis were performed using HUMAnN 2 (version 2.8.1) (54), respectively. Shannon indices of α diversity, PCoA analysis with Bray-Curtis distance, and taxonomic composition analysis were performed using R package microbiome (version 1.21.0). The LEfSe analysis was performed by the docker version of LEfSe (version 1.0.0) (46) with the LDA cutoff set as 2.

### Plasma and colon-content sample preparation and extraction

Mice were euthanized by cervical dislocation at the end of behavior tests. Plasma and cecum content were collected and transferred to a −80°C refrigerator. The sample stored at −80°C refrigerator was thawed on ice and vortexed for 10 s. A 150 µL extract solution (ACN: methanol = 1:4, vol/vol) containing internal standard was added to a 50 µL sample. Then, the sample was vortexed for 3 min and centrifuged at 12,000 rpm for 10 min (4°C). A 150 µL aliquots of the supernatant were collected and placed in −20°C for 30 min, and then centrifuged at 12,000 rpm for 3 min (4°C). Aliquots of the supernatant at 120 µL were transferred for LC-MS analysis.

### T3 UPLC conditions

The sample extracts were analyzed using an LC-ESI-MS/MS system (UPLC, ExionLC AD,ˈhttps://sciex.com.cn/; MS, QTRAP System, https://sciex.com/). The analytical conditions were as follows: UPLC: column, Waters ACQUITY UPLC HSS T3 C18 (1.8 µm, 2.1 mm*100 mm); column temperature, 40°C; flow rate, 0.4 mL/min; injection volume, 2 or 5 µL; solvent system, water (0.1% formic acid):acetonitrile (0.1% formic acid); gradient program, 95:5 vol/vol at 0 min, 10:90 vol/vol at 10.0 min, 10:90 vol/vol at 11.0 min, 95:5 vol/vol at 11.1 min, 95:5 vol/vol at 14.0 min.

### QTOF-MS/MS

The Triple TOF mass spectrometer was used for its ability to acquire MS/MS spectra on an information-dependent basis (IDA) during an LC/MS experiment. In this mode, the acquisition software (TripleTOF 6600, AB SCIEX) continuously evaluates the full scan survey MS data as it collects and triggers the acquisition of MS/MS spectra depending on preselected criteria. In each cycle, 12 precursor ions whose intensity is greater than 100 were chosen for fragmentation at collision energy (CE) of 30 V (12 MS/MS events with product ion accumulation time of 50 ms each). ESI source conditions were set as follows: ion source gas 1 as 50 psi, ion source gas 2 as 50 psi, curtain gas as 25 psi, source temperature 500°C, ion spray voltage floating (ISVF) 5,500 V or −4,500 V in positive or negative mode, respectively.

### ESI-Q TRAP-MS/MS

LIT and triple quadrupole (QQQ) scans were acquired on a triple quadrupole-linear ion trap mass spectrometer (QTRAP), QTRAP LC-MS/MS System, equipped with an ESI Turbo Ion-Spray interface, operating in positive and negative ion mode and controlled by Analyst 1.6.3 software (Sciex). The ESI source operation parameters were as follows: source temperature 500°C; ion spray voltage (IS) 5,500 V (positive), −4,500 V (negative); ion source gas I (GSI), gas II (GSII), curtain gas (CUR) was set at 50, 50, and 25.0 psi, respectively; the collision gas (CAD) was high. Instrument tuning and mass calibration were performed with 10 and 100 µmol/L polypropylene glycol solutions in QQQ and LIT modes, respectively. A specific set of MRM transitions was monitored for each period according to the metabolites eluted within this period.

### Metabolomics analysis

The qualitative and quantitative determination of metabolites was conducted utilizing both a proprietary database, MWDB (Metware Biotechnology Co., Ltd., Wuhan, China), and publicly accessible databases, including the Human Metabolome Database (HMDB; https://www.hmdb.ca), MassBank (https://www.massbank.jp), METLIN (https://metlin.scripps.edu), KEGG (https://www.kegg.jp), MoNA (https://mona.fiehnlab.ucdavis.edu), PubChem (https://pubchem.ncbi.nlm.nih.gov), and ChemSpider (https://www.chemspider.com). The annotated peak abundance tables from plasma metabolomics and colon-content metabolomics were uploaded to MetaboAnalyst 5 (57) (https://www.metaboanalyst.ca/MetaboAnalyst/) for data pre-processing (normalization by sum, log transformation, and autoscaling) and downstream analysis: principal component analysis (PCA), differential analysis and pathway analysis. 3D PCA was generated additionally using the R package scatterplot3d (version 0.3-44). Metabolites exhibiting a VIP (variable importance in projection) value >1, a *P* < 0.05, and a fold change >2 or <0.5 were identified as differentially expressed between the two groups. The volcano plots were generated based on the log_2_(fold change), −log_10_(*P*), and VIP values of the metabolites. The pathway analysis was performed based on the *Mus musculus* (KEGG) pathway library on the MetaboAnalyst (57) website. The taxonomic classes of chemical compounds were annotated by R package biodbHMDB (version 1.8.0) and Class II was selected and manually curated for final visualization. All other statistical analyses and plotting were performed in R (version 4.2.1).

### Integration of multi-omics data

Samples from the BTBR+FMT group and the BTBR+Saline group were used to calculate the correlation. The correlation of metabolite abundance, microbial species level, and social behavior index was calculated using Spearman’s rank correlation method across the BTBR mice samples. The *P* value was corrected by the false positive rate (FDR) method using R package psych package (version 2.4.1) (105). All statistical analyses and plotting were performed in R (version 4.2.1).

WGCNA analysis (version 1.72-5) (106) was performed to construct significant modules in each omics data. We optimized the soft threshold to 10 for the metagenomics data, 14 for the plasma metabolomics data, and 12 for the colon-content metabolomics data. The features were clustered into modules by hierarchical clustering with Euclidian distance, and similar modules were further merged. The correlation within modules and correlation between modules and social behavior index was also measured by Pearson correlation using raw *P* value < 0.05. Modules that were associated with the social behavior index were explored for potential patterns of compound classes or taxa compositions.

A paired metabolite abundance table and metagenomics profile table were uploaded to MetOrigin (https://metorigin.met-bioinformatics.cn/) for metabolite origin tracking analysis. The analysis type was set as “Deep MetOrigin Analysis,” the host was set as “Mus musculus (house mouse).” For both metagenomics and metabolomics data, the test method “Auto” was selected, and *P* value cutoff was set to 0.05. Sankey diagram and network between metabolites and microbes were generated for metabolites belonging to the host, bacteria, and both (co-metabolism).

## Data Availability

The raw sequencing data have been deposited in NCBI BioProject database under BioProject accession no. PRJNA1047191. All data analyzed during this study are included in this article and deposited in the Zenodo (DOI: 10.5281/zenodo.10974729).

## SUPPLEMENTAL MATERIAL

Fig. S1 msystems.00257-24-s0001.pdf Effects of human FMT and vitamin B6 on the anxiety-like and stereotypic behavior of the BTBR mice.

Fig. S2 msystems.00257-24-s0002.pdf Gut microbiota and metabolomics differ between BTBR + saline and FMT groups.

Fig. S3 msystems.00257-24-s0003.pdf Metagenomic pathways altered by FMT treatment and abundance information of Bacteroides species present in the PLP biosynthesis I pathway.

Fig. S4 msystems.00257-24-s0004.pdf Correlation analysis between differential microbes/metabolites and the BTBR mouse behaviors.

Fig. S5 msystems.00257-24-s0005.pdf Correlation analysis between differential microbes/metabolites from the colon-content metagenomics, colon-content metabolomics, and plasma metabolomics.

Fig. S6 msystems.00257-24-s0006.pdf Network analysis of paired colon-content metagenomics and colon-content metabolomics data using MetOrigin.

Fig. S7 msystems.00257-24-s0007.pdf Network analysis of paired colon-content metagenomics and plasma metabolomics data using MetOrigin.

Table S1 msystems.00257-24-s0008.xlsx The taxonomic compositions of samples in donor, the BTBR + saline, and BTBR + FMT group.

Table S2 msystems.00257-24-s0009.xlsx Pathway analysis of colon-content and plasma metabolomics between BTBR + saline group and BTBR + FMT group.

Table S3 msystems.00257-24-s0010.xlsx WGCNA modules extracted from metagenomics data set (MGS), colon-content metabolomics (MIC), and plasma metabolomics (PL).

ASM does not own the copyrights to Supplemental Material that may be linked to, or accessed through, an article. The authors have granted ASM a non-exclusive, world-wide license to publish the Supplemental Material files. Please contact the corresponding author directly for reuse.
