# Supplementary material for: Human-derived fecal microbiota transplantation alleviates social deficits of the BTBR mouse model of autism through a potential mechanism involving vitamin B6 metabolism
Source: mSystems. 2024 May 23;9(6):e00257-24. doi: 10.1128/msystems.00257-24 (PMC11237617; doi:10.1128/msystems.00257-24)
Supplement: Fig. S1 — Effects of human FMT and vitamin B6 on the anxiety-like and stereotypic behavior of the BTBR mice. [file msystems.00257-24-s0001.pdf]

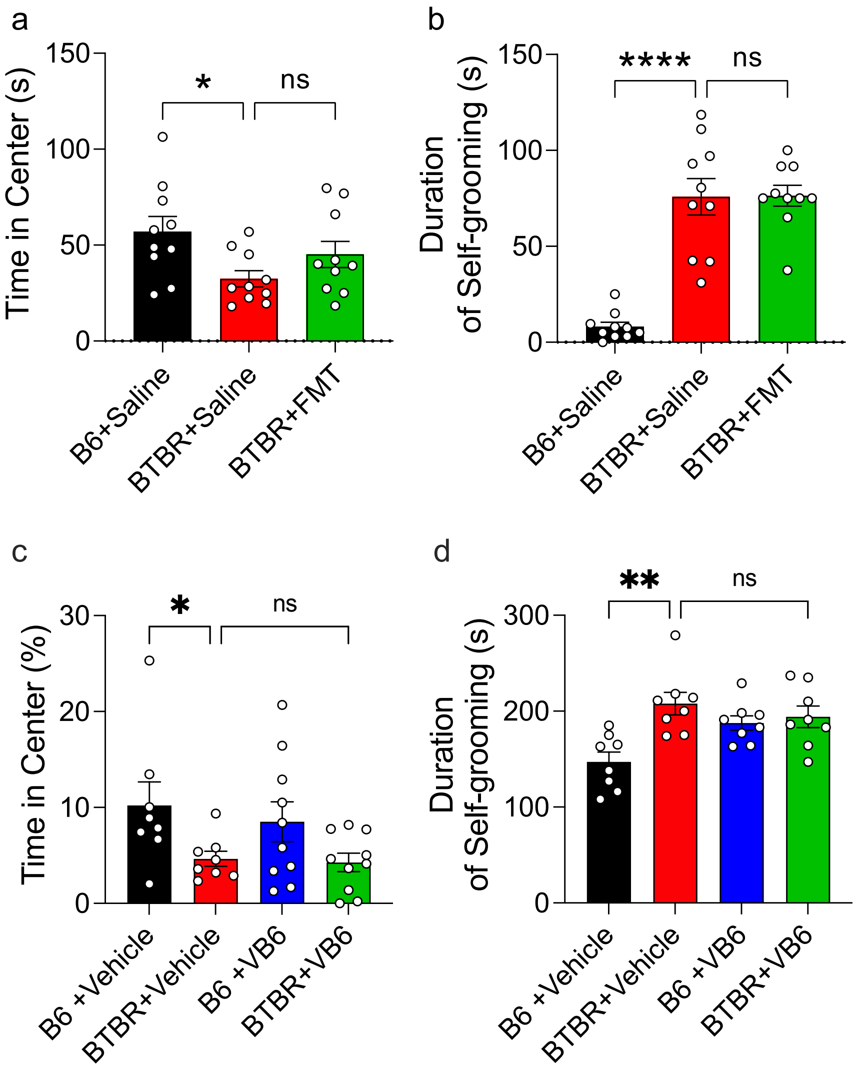

a
Time in Center (s)
150
100
50
0
*
ns
B6+Saline
BTBR+Saline
BTBR+FMT
b
Duration of Self-grooming (s)
150
100
50
0
****
ns
B6+Saline
BTBR+Saline
BTBR+FMT
c
Time in Center (%)
30
20
10
0
*
ns
B6 +Vehicle
BTBR+Vehicle
B6 +VB6
BTBR+VB6
d
Duration of Self-grooming (s)
300
200
100
0
**
ns
B6 +Vehicle
BTBR+Vehicle
B6 +VB6
BTBR+VB6

Figure S1. Effects of human FMT and vitamin B_6 on the anxiety-like and stereotypic behavior of the BTBR mice.

a The percentage of the time mice spent in the center of the open field test box. **b** Duration time of self-grooming in open field test. **c** The percentage of the time mice spent in the center of the open field test box. **d** Duration time of self-grooming in open field test. Data represent the mean ± SEM. Error bars indicate SEM. * $P < 0.05$, **** P < 0.0001, *ns* no significance. The test used in a-d was two-way ANOVA with Tukey's post hoc test.
