# Supplementary material for: Human-derived fecal microbiota transplantation alleviates social deficits of the BTBR mouse model of autism through a potential mechanism involving vitamin B6 metabolism
Source: mSystems. 2024 May 23;9(6):e00257-24. doi: 10.1128/msystems.00257-24 (PMC11237617; doi:10.1128/msystems.00257-24)
Supplement: Fig. S2 — Gut microbiota and metabolomics differ between BTBR + saline and FMT groups. [file msystems.00257-24-s0002.pdf]

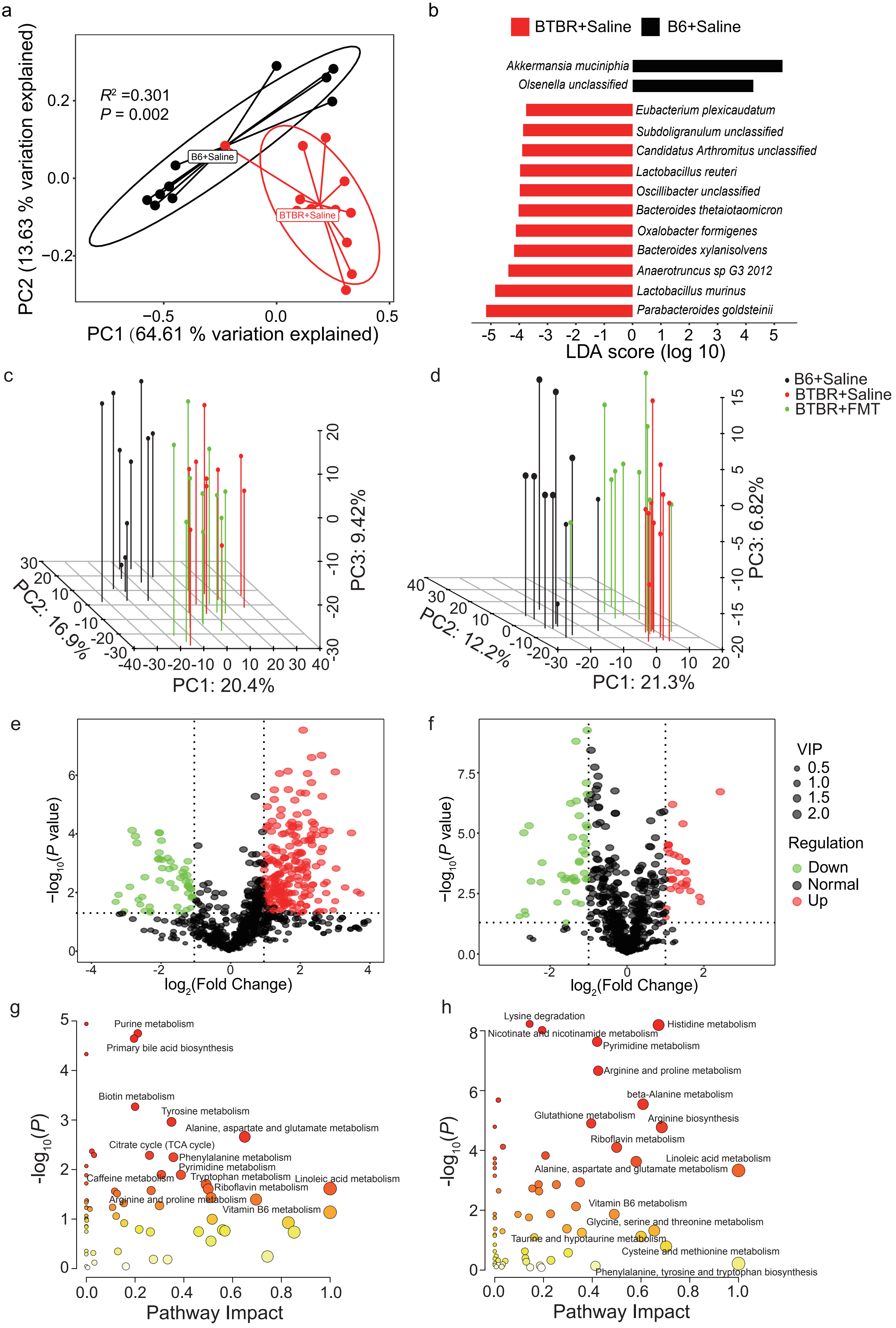

a
R^2 =0.301
P = 0.002
B6+Saline
BTBR+Saline
PC1 (64.61 % variation explained)
PC2 (13.63 % variation explained)
b
BTBR+Saline
B6+Saline
Akkermansia muciniphia
Olsenella unclassified
Eubacterium plexicaudatum
Subdoligranulum unclassified
Candidatus Arthromitus unclassified
Lactobacillus reuteri
Oscillibacter unclassified
Bacteroides thetaiotaomicron
Oxalobacter formigenes
Bacteroides xylanisolvens
Anaerotruncus sp G3 2012
Lactobacillus murinus
Parabacteroides goldsteinii
LDA score (log 10)
c
PC1: 20.4%
PC2: 16.9%
PC3: 9.42%
d
PC1: 21.3%
PC2: 12.2%
PC3: 6.82%
B6+Saline
BTBR+Saline
BTBR+FMT
e
f
$-\log_{10}(P$ value)
$\log_2$(Fold Change)
VIP
0.5
1.0
1.5
2.0
Regulation
Down
Normal
Up
g
Purine metabolism
Primary bile acid biosynthesis
Biotin metabolism
Tyrosine metabolism
Alanine, aspartate and glutamate metabolism
Citrate cycle (TCA cycle)
Phenylalanine metabolism
Pyrimidine metabolism
Caffeine metabolism
Tryptophan metabolism
Linoleic acid metabolism
Riboflavin metabolism
Arginine and proline metabolism
Vitamin B6 metabolism
$-\log_{10}(P)$
Pathway Impact
h
Lysine degradation
Histidine metabolism
Nicotinate and nicotinamide metabolism
Pyrimidine metabolism
Arginine and proline metabolism
beta-Alanine metabolism
Glutathione metabolism
Arginine biosynthesis
Riboflavin metabolism
Linoleic acid metabolism
Alanine, aspartate and glutamate metabolism
Vitamin B6 metabolism
Glycine, serine and threonine metabolism
Taurine and hypotaurine metabolism
Cysteine and methionine metabolism
Phenylalanine, tyrosine and tryptophan biosynthesis
$-\log_{10}(P)$
Pathway Impact

Figure S2. Gut microbiota and metabolomics differ between BTBR + Saline and FMT groups.

a PCoA analysis based on the Bray-Curtis distance between the BTBR + Saline and B6 + Saline groups. PERMANOVA test was applied for the *p*-value. **b** LEfSe analysis showing the differential microbes between the BTBR + Saline and B6 + Saline groups (LDA cutoff set as 2.0). **c** PCA analysis of colon-content samples from the B6 + Saline, BTBR + Saline and BTBR + FMT groups. **d** PCA analysis of plasma samples from the B6 + Saline, BTBR + Saline and BTBR + FMT groups. **e** The volcano plot illustrating the differential colon-content metabolites between the BTBR + Saline group and the B6 + Saline group. **f** The volcano plot illustrating the differential plasma metabolites between the BTBR + Saline group and the B6 + Saline group. The legend of **e** and **f** is shown on the right. The dots represented in green (down-regulated) and red (up-regulated) are differentially regulated metabolites with $|\log_2(\text{fold change})| > 1$, a P value < 0.05, and VIP value > 1.0. VIP value represents the importance projection value of the metabolite obtained from the PLS-DA model. **g** Bubble plot of altered metabolic pathways between the BTBR + Saline group and the B6 + Saline group using the colon-content metabolomics data. **h** Bubble plot of altered metabolic pathways between the BTBR + Saline group and the B6 + Saline group using the plasma metabolomics data. The bubble size corresponds to the pathway impact with a color gradient denoting the significance from the highest (red) to the lowest (white).
