# Supplementary material for: Human-derived fecal microbiota transplantation alleviates social deficits of the BTBR mouse model of autism through a potential mechanism involving vitamin B6 metabolism
Source: mSystems. 2024 May 23;9(6):e00257-24. doi: 10.1128/msystems.00257-24 (PMC11237617; doi:10.1128/msystems.00257-24)
Supplement: Fig. S3 — Metagenomic pathways altered by FMT treatment and abundance information of Bacteroides species present in the PLP biosynthesis I pathway. [file msystems.00257-24-s0003.pdf]

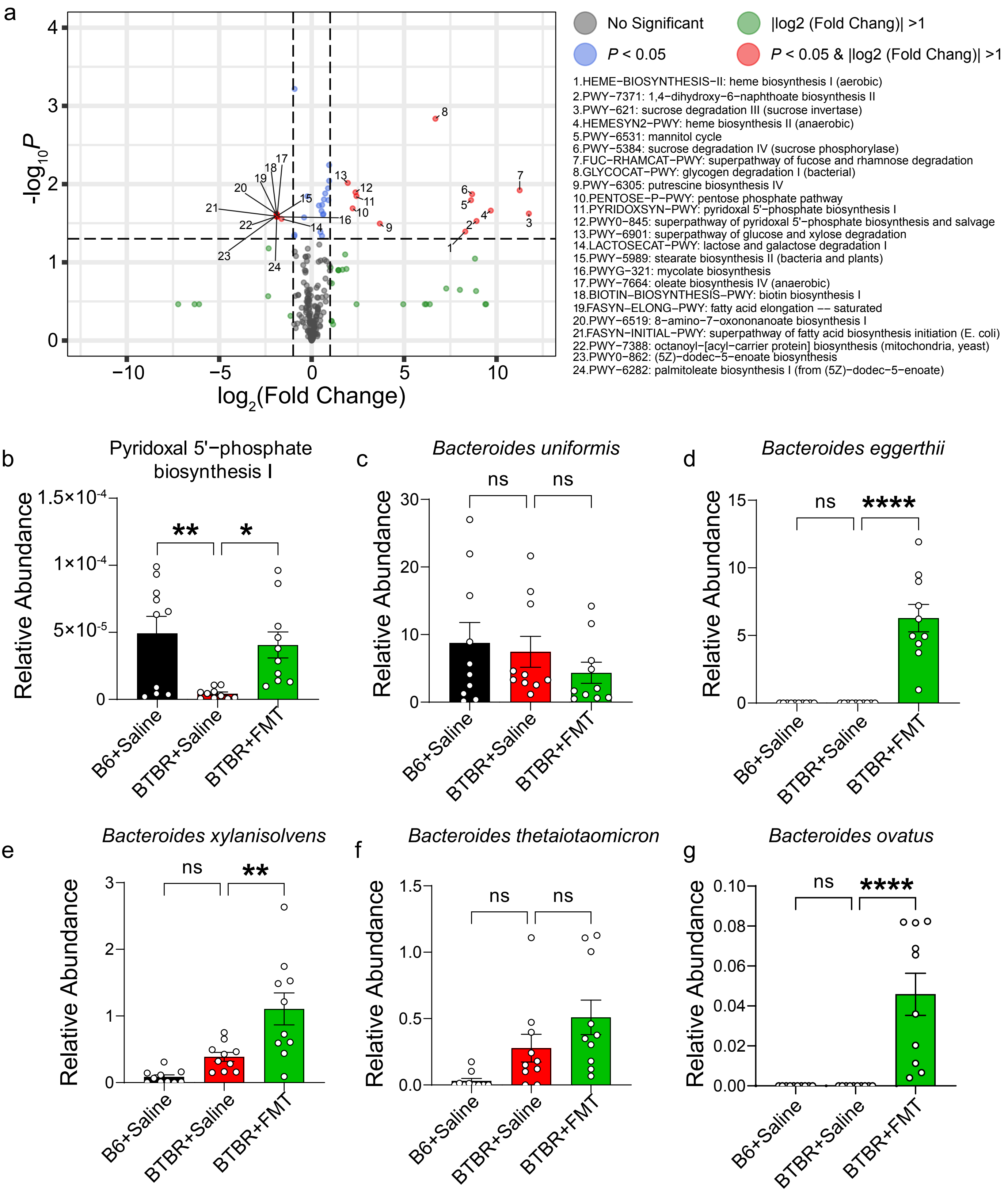

a
No Significant
|log2 (Fold Chang)| >1
P < 0.05
P < 0.05 & |log2 (Fold Chang)| >1
1.HEME−BIOSYNTHESIS−II: heme biosynthesis I (aerobic)
2.PWY−7371: 1,4−dihydroxy−6−naphthoate biosynthesis II
3.PWY−621: sucrose degradation III (sucrose invertase)
4.HEMESYN2−PWY: heme biosynthesis II (anaerobic)
5.PWY−6531: mannitol cycle
6.PWY−5384: sucrose degradation IV (sucrose phosphorylase)
7.FUC−RHAMCAT−PWY: superpathway of fucose and rhamnose degradation
8.GLYCOCAT−PWY: glycogen degradation I (bacterial)
9.PWY−6305: putrescine biosynthesis IV
10.PENTOSE−P−PWY: pentose phosphate pathway
11.PYRIDOXSYN−PWY: pyridoxal 5'−phosphate biosynthesis I
12.PWY0−845: superpathway of pyridoxal 5'−phosphate biosynthesis and salvage
13.PWY−6901: superpathway of glucose and xylose degradation
14.LACTOSECAT−PWY: lactose and galactose degradation I
15.PWY−5989: stearate biosynthesis II (bacteria and plants)
16.PWYG−321: mycolate biosynthesis
17.PWY−7664: oleate biosynthesis IV (anaerobic)
18.BIOTIN−BIOSYNTHESIS−PWY: biotin biosynthesis I
19.FASYN−ELONG−PWY: fatty acid elongation −− saturated
20.PWY−6519: 8−amino−7−oxononanoate biosynthesis I
21.FASYN−INITIAL−PWY: superpathway of fatty acid biosynthesis initiation (E. coli)
22.PWY−7388: octanoyl−[acyl−carrier protein] biosynthesis (mitochondria, yeast)
23.PWY0−862: (5Z)−dodec−5−enoate biosynthesis
24.PWY−6282: palmitoleate biosynthesis I (from (5Z)−dodec−5−enoate)
-log10P
log2(Fold Change)
b
Pyridoxal 5'−phosphate biosynthesis I
c
Bacteroides uniformis
d
Bacteroides eggerthii
e
Bacteroides xylanisolvens
f
Bacteroides thetaiotaomicron
g
Bacteroides ovatus
Relative Abundance
B6+Saline
BTBR+Saline
BTBR+FMT
ns
**
*
****

Figure S3. Metagenomic pathways altered by FMT treatment and abundance information of *Bacteroides* species present in the PLP biosynthesis I pathway.

a Volcano plot showing altered metagenomic pathways between the BTBR + FMT and BTBR + Saline groups. **b** Relative abundance of pyridoxal 5'−phosphate biosynthesis I pathway across different groups. **c-g** Abundance of *B. uniformis*, *B. eggerthii*, *B. xylanisolvens*, *B. thetaiotaomicron* and *B. ovatus* across the B6 + Saline, BTBR + Saline and BTBR + FMT groups. Data represent the mean ± SEM. Error bars indicate SEM. * $P < 0.05$, ** $P < 0.01$, **** $P < 0.0001$, *ns* no significance. The test used in **b-g** was one-way ANOVA with Tukey's post hoc test.
