# Supplementary material for: Human-derived fecal microbiota transplantation alleviates social deficits of the BTBR mouse model of autism through a potential mechanism involving vitamin B6 metabolism
Source: mSystems. 2024 May 23;9(6):e00257-24. doi: 10.1128/msystems.00257-24 (PMC11237617; doi:10.1128/msystems.00257-24)
Supplement: Fig. S4 — Correlation analysis between differential microbes/metabolites and the BTBR mouse behaviors. [file msystems.00257-24-s0004.pdf]

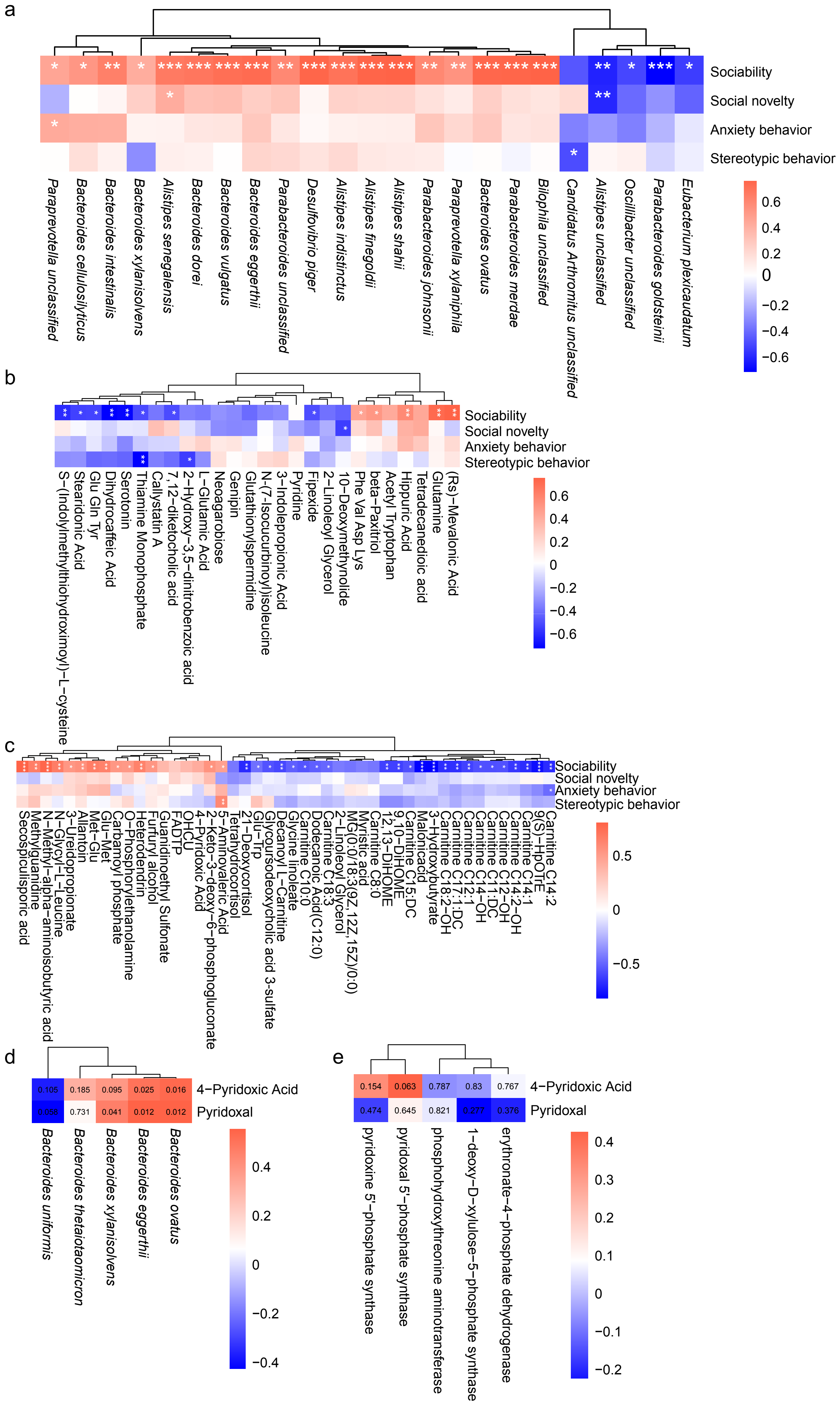

a
Sociability
Social novelty
Anxiety behavior
Stereotypic behavior
Paraprevotella unclassified
Bacteroides cellulosilyticus
Bacteroides intestinalis
Bacteroides xylanisolvens
Alistipes senegalensis
Bacteroides dorei
Bacteroides vulgatus
Bacteroides eggerthii
Parabacteroides unclassified
Desulfovibrio piger
Alistipes indistinctus
Alistipes finegoldii
Alistipes shahii
Parabacteroides johnsonii
Paraprevotella xylaniphila
Bacteroides ovatus
Parabacteroides merdae
Bilophila unclassified
Candidatus Arthromitus unclassified
Alistipes unclassified
Oscillibacter unclassified
Parabacteroides goldsteinii
Eubacterium plexicaudatum
0.6
0.4
0.2
0
−0.2
−0.4
−0.6
b
Sociability
Social novelty
Anxiety behavior
Stereotypic behavior
S−(Indolylmethylthiohydroximoyl)−L−cysteine
Stearidonic Acid
Glu Gln Tyr
Dihydrocaffeic Acid
Serotonin
Thiamine Monophosphate
Callystatin A
7,12−diketocholic acid
2−Hydroxy−3,5−dinitrobenzoic acid
L−Glutamic Acid
Neoagarobiose
Genipin
Glutathionylspermidine
N−(7−Isocucurbinoyl)isoleucine
3−Indolepropionic Acid
Pyridine
Fipexide
2−Linoleoyl Glycerol
10−Deoxymethynolide
Phe Val Asp Lys
beta−Paxitriol
Acetyl Tryptophan
Hippuric Acid
Tetradecanedioic acid
Glutamine
(Rs)−Mevalonic Acid
0.6
0.4
0.2
0
−0.2
−0.4
−0.6
c
Sociability
Social novelty
Anxiety behavior
Stereotypic behavior
Secospiculisporic acid
Methylguanidine
N−Methyl−alpha−aminoisobutyric acid
N−Glycyl−L−Leucine
3−Ureidopropionate
Allantoin
Met−Glu
Glu−Met
Carbamoyl phosphate
O−Phosphorylethanolamine
Heterodendrin
Furfuryl alcohol
Guanidinoethyl Sulfonate
FADTP
OHCU
4−Pyridoxic Acid
2−Keto−3−deoxy−6−phosphogluconate
5−Aminovaleric Acid
Tetrahydrocortisol
21−Deoxycortisol
Glu−Trp
Glycoursodeoxycholic acid 3−sulfate
Decanoyl L−Carnitine
Glycine linoleate
Carnitine C10:0
Dodecanoic Acid(C12:0)
Carnitine C18:3
2−Linoleoyl Glycerol
MG(0:0/18:3(9Z,12Z,15Z)/0:0)
Myristic acid
Carnitine C8:0
12,13−DiHOME
9,10−DiHOME
Carnitine C15:DC
Malonicacid
3−Hydroxybutyrate
Carnitine C18:2−OH
Carnitine C17:1:DC
Carnitine C12:1
Carnitine C14−OH
Carnitine C11:DC
Carnitine C12−OH
Carnitine C14:2−OH
Carnitine C14:1
9(S)−HpOTrE
Carnitine C14:2
0.5
0
−0.5
d
4−Pyridoxic Acid
Pyridoxal
0.105 0.185 0.095 0.025 0.016
0.068 0.731 0.041 0.012 0.012
Bacteroides uniformis
Bacteroides thetaiotaomicron
Bacteroides xylanisolvens
Bacteroides eggerthii
Bacteroides ovatus
0.4
0.2
0
−0.2
−0.4
e
4−Pyridoxic Acid
Pyridoxal
0.154 0.063 0.787 0.83 0.767
0.474 0.645 0.821 0.277 0.376
pyridoxine 5'−phosphate synthase
pyridoxal 5'−phosphate synthase
phosphohydroxythreonine aminotransferase
1−deoxy−D−xylulose−5−phosphate synthase
erythronate−4−phosphate dehydrogenase
0.4
0.3
0.2
0.1
0
−0.1
−0.2

Figure S4. Correlation analysis between differential microbes/metabolites and the BTBR mouse behaviors.

a Correlation between the differential microbes and mouse behaviors across the BTBR mouse samples. **b** Correlation between the differential colon-content metabolites and the mouse behaviors across the BTBR mouse samples. **c** Correlation between the differential plasma metabolites and the mouse behaviors. **d** Correlation between *Bacteroides* spp. present in PLP biosynthesis I pathway and metabolites involved in vitamin B_6 metabolism. **e** Correlation between enzymes in the PLP biosynthesis I pathway and metabolites involved in vitamin B_6 metabolism. Spearman's Rank Correlation was used for the correlation coefficients. For **a**-**c**, Benjamini-Hochberg (BH) adjusted p values for significance are noted. $*P < 0.05$, $**P < 0.01$, $***P < 0.001$. The color scale denotes Spearman's r from red (positive correlation) to blue (negative correlation).
