# Supplementary material for: Human-derived fecal microbiota transplantation alleviates social deficits of the BTBR mouse model of autism through a potential mechanism involving vitamin B6 metabolism
Source: mSystems. 2024 May 23;9(6):e00257-24. doi: 10.1128/msystems.00257-24 (PMC11237617; doi:10.1128/msystems.00257-24)
Supplement: Fig. S5 — Correlation analysis between differential microbes/metabolites from the colon-content metagenomics, colon-content metabolomics, and plasma metabolomics. [file msystems.00257-24-s0005.pdf]

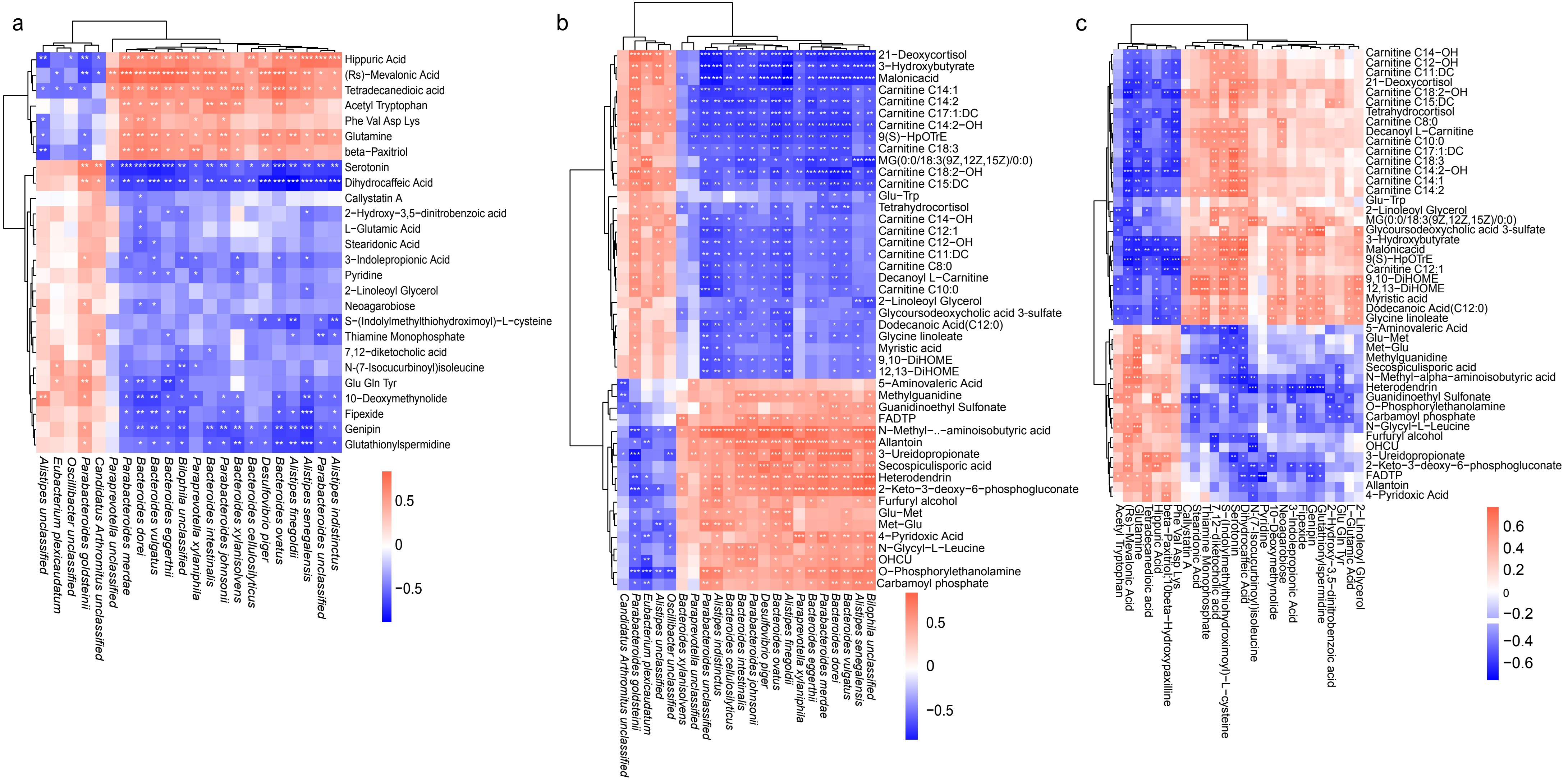
a
Hippuric Acid
(Rs)−Mevalonic Acid
Tetradecanedioic acid
Acetyl Tryptophan
Phe Val Asp Lys
Glutamine
beta−Paxitriol
Serotonin
Dihydrocaffeic Acid
Callystatin A
2−Hydroxy−3,5−dinitrobenzoic acid
L−Glutamic Acid
Stearidonic Acid
3−Indolepropionic Acid
Pyridine
2−Linoleoyl Glycerol
Neoagarobiose
S−(Indolylmethylthiohydroximoyl)−L−cysteine
Thiamine Monophosphate
7,12−diketocholic acid
N−(7−Isocucurbinoyl)isoleucine
Glu Gln Tyr
10−Deoxymethynolide
Fipexide
Genipin
Glutathionylspermidine
Alistipes unclassified
Eubacterium plexicaudatum
Oscillibacter unclassified
Parabacteroides goldsteinii
Candidatus Arthromitus unclassified
Paraprevotella unclassified
Parabacteroides merdae
Bacteroides dorei
Bacteroides vulgatus
Bacteroides eggerthii
Bilophila unclassified
Paraprevotella xylaniphila
Bacteroides intestinalis
Parabacteroides johnsonii
Bacteroides xylanisolvens
Bacteroides cellulosilyticus
Desulfovibrio piger
Bacteroides ovatus
Alistipes finegoldii
Alistipes senegalensis
Parabacteroides unclassified
Alistipes indistinctus
0.5
0
−0.5
b
21−Deoxycortisol
3−Hydroxybutyrate
Malonicacid
Carnitine C14:1
Carnitine C14:2
Carnitine C17:1:DC
Carnitine C14:2−OH
9(S)−HpOTrE
Carnitine C18:3
MG(0:0/18:3(9Z,12Z,15Z)/0:0)
Carnitine C18:2−OH
Carnitine C15:DC
Glu−Trp
Tetrahydrocortisol
Carnitine C14−OH
Carnitine C12:1
Carnitine C12−OH
Carnitine C11:DC
Carnitine C8:0
Decanoyl L−Carnitine
Carnitine C10:0
2−Linoleoyl Glycerol
Glycoursodeoxycholic acid 3-sulfate
Dodecanoic Acid(C12:0)
Glycine linoleate
Myristic acid
9,10−DiHOME
12,13−DiHOME
5−Aminovaleric Acid
Methylguanidine
Guanidinoethyl Sulfonate
FADTP
N−Methyl−..−aminoisobutyric acid
Allantoin
3−Ureidopropionate
Secospiculisporic acid
Heterodendrin
2−Keto−3−deoxy−6−phosphogluconate
Furfuryl alcohol
Glu−Met
Met−Glu
4−Pyridoxic Acid
N−Glycyl−L−Leucine
OHCU
O−Phosphorylethanolamine
Carbamoyl phosphate
Candidatus Arthromitus unclassified
Parabacteroides goldsteinii
Eubacterium plexicaudatum
Alistipes unclassified
Oscillibacter unclassified
Bacteroides xylanisolvens
Paraprevotella unclassified
Parabacteroides unclassified
Alistipes indistinctus
Bacteroides cellulosilyticus
Bacteroides intestinalis
Parabacteroides johnsonii
Desulfovibrio piger
Bacteroides ovatus
Alistipes finegoldii
Paraprevotella xylaniphila
Bacteroides eggerthii
Parabacteroides merdae
Bacteroides dorei
Bacteroides vulgatus
Alistipes senegalensis
Bilophila unclassified
0.5
0
−0.5
c
Carnitine C14−OH
Carnitine C12−OH
Carnitine C11:DC
21−Deoxycortisol
Carnitine C18:2−OH
Carnitine C15:DC
Tetrahydrocortisol
Carnitine C8:0
Decanoyl L−Carnitine
Carnitine C10:0
Carnitine C17:1:DC
Carnitine C18:3
Carnitine C14:2−OH
Carnitine C14:1
Carnitine C14:2
Glu−Trp
2−Linoleoyl Glycerol
MG(0:0/18:3(9Z,12Z,15Z)/0:0)
Glycoursodeoxycholic acid 3-sulfate
3−Hydroxybutyrate
Malonicacid
9(S)−HpOTrE
Carnitine C12:1
9,10−DiHOME
12,13−DiHOME
Myristic acid
Dodecanoic Acid(C12:0)
Glycine linoleate
5−Aminovaleric Acid
Glu−Met
Met−Glu
Methylguanidine
Secospiculisporic acid
N−Methyl−alpha−aminoisobutyric acid
Heterodendrin
Guanidinoethyl Sulfonate
O−Phosphorylethanolamine
Carbamoyl phosphate
N−Glycyl−L−Leucine
Furfuryl alcohol
OHCU
3−Ureidopropionate
2−Keto−3−deoxy−6−phosphogluconate
FADTP
Allantoin
4−Pyridoxic Acid
Acetyl Tryptophan
(Rs)−Mevalonic Acid
Glutamine
Tetradecanedioic acid
Hippuric Acid
beta−Paxitriol;10beta−Hydroxypaxilline
Phe Val Asp Lys
Callystatin A
Stearidonic Acid
Thiamine Monophosphate
7,12−diketocholic acid
S−(Indolylmethylthiohydroximoyl)−L−cysteine
Serotonin
Dihydrocaffeic Acid
N−(7−Isocucurbinoyl)isoleucine
Pyridine
10−Deoxymethynolide
Neoagarobiose
3−Indolepropionic Acid
Fipexide
Genipin
Glutathionylspermidine
2−Hydroxy−3,5−dinitrobenzoic acid
Glu Gln Tyr
L−Glutamic Acid
2−Linoleoyl Glycerol
0.6
0.4
0.2
0
−0.2
−0.4
−0.6

Figure S5. Correlation analysis between differential microbes/metabolites from the colon-content metagenomics, colon-content metabolomics, and plasma metabolomics.

a Correlation between the differential colon-content metabolites and microbes across the BTBR mouse samples. b Correlation between the differential plasma metabolites and microbes across the BTBR mouse samples. **c** Correlation between the differential colon-content metabolites and plasma metabolites. Spearman's Rank Correlation was used for the correlation coefficients. False discovery rate (FDR)-adjusted *p* values for significance are noted. *$P < 0.05$, **$P < 0.01$, ***$P < 0.001$. The color scale denotes Spearman's r from red (positive correlation) to blue (negative correlation).
