# Supplementary material for: Human-derived fecal microbiota transplantation alleviates social deficits of the BTBR mouse model of autism through a potential mechanism involving vitamin B6 metabolism
Source: mSystems. 2024 May 23;9(6):e00257-24. doi: 10.1128/msystems.00257-24 (PMC11237617; doi:10.1128/msystems.00257-24)
Supplement: Fig. S6 — Network analysis of paired colon-content metagenomics and colon-content metabolomics data using MetOrigin. [file msystems.00257-24-s0006.pdf]

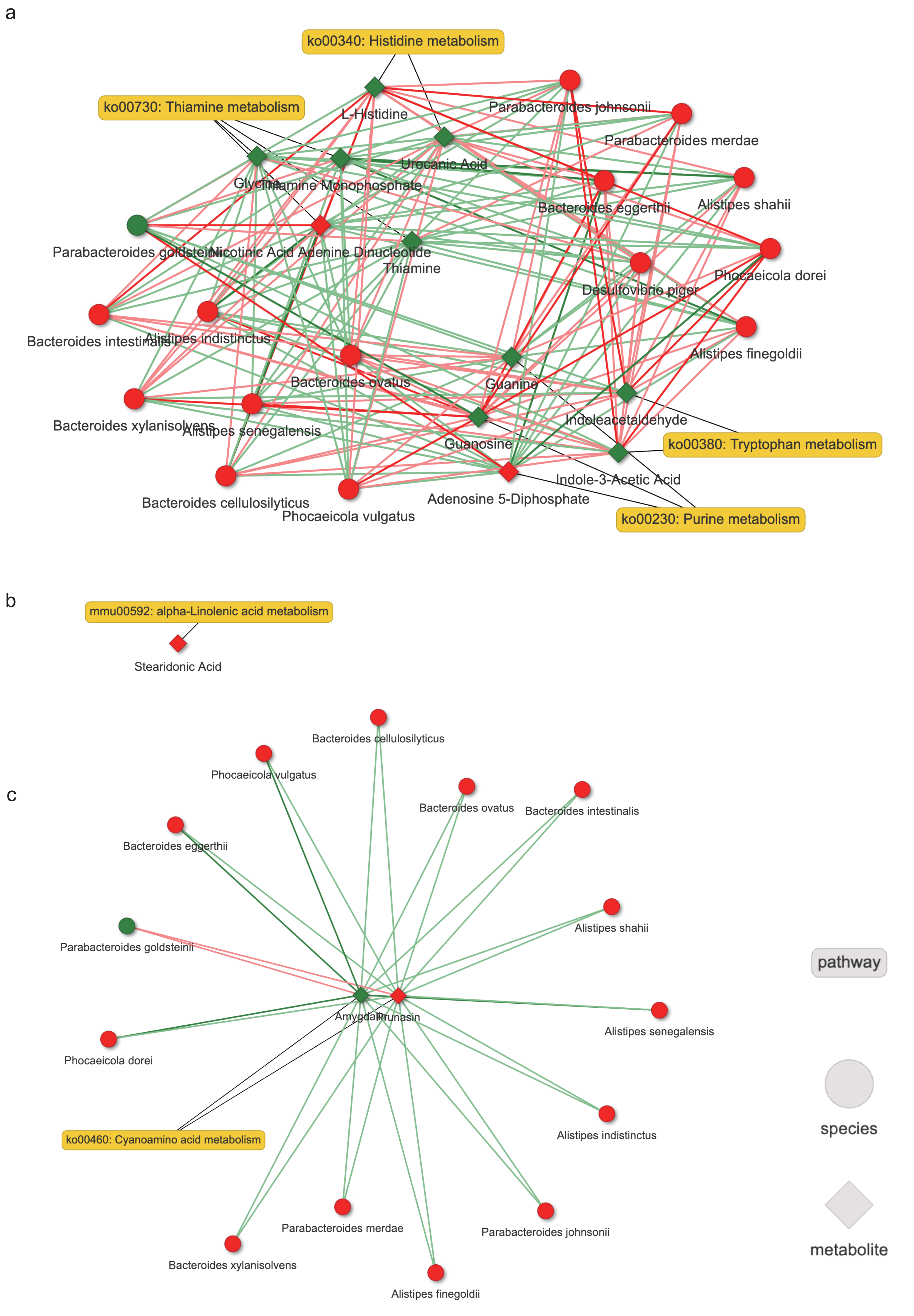
a
ko00340: Histidine metabolism
ko00730: Thiamine metabolism
L-Histidine
Urocanic Acid
Glycine
Thiamine Monophosphate
Nicotinic Acid Adenine Dinucleotide
Thiamine
Parabacteroides johnsonii
Parabacteroides merdae
Bacteroides eggerthii
Alistipes shahii
Phocaeicola dorei
Desulfovibrio piger
Alistipes finegoldii
Parabacteroides goldsteinii
Bacteroides intestinalis
Alistipes indistinctus
Bacteroides ovatus
Guanine
Indoleacetaldehyde
Bacteroides xylanisolvens
Alistipes senegalensis
Guanosine
ko00380: Tryptophan metabolism
Indole-3-Acetic Acid
Adenosine 5-Diphosphate
Bacteroides cellulosilyticus
Phocaeicola vulgatus
ko00230: Purine metabolism
b
mmu00592: alpha-Linolenic acid metabolism
Stearidonic Acid
c
Bacteroides cellulosilyticus
Phocaeicola vulgatus
Bacteroides ovatus
Bacteroides intestinalis
Bacteroides eggerthii
Alistipes shahii
Parabacteroides goldsteinii
Amygdalin
Prunasin
Alistipes senegalensis
Phocaeicola dorei
Alistipes indistinctus
ko00460: Cyanoamino acid metabolism
Parabacteroides merdae
Parabacteroides johnsonii
Bacteroides xylanisolvens
Alistipes finegoldii
pathway
species
metabolite

Figure S6. Network analysis of paired colon-content metagenomics and colon-content metabolomics data using MetOrigin.

a Co-metabolism network connecting differential metabolites shared by the host and microbiota to the related microbes. **b** Host network shows interactions between differential metabolites with host origin. **c** Microbiota network shows interactions between differential microbial-specific metabolites and related microbes. The interaction (edge) integrates statistical (STA) and biological (BIO) significance. Node shape: rectangle indicates pathway name, diamond indicates metabolite and dot denotes microbe. Node color: red indicates upregulated metabolite or microbe, and green indicates downregulated metabolite or microbe. The node of deep color denotes statistical significance ($P < 0.05$), while the node of light color indicates no significance found ($P \geq 0.05$). Node edge: red indicates positive correlations ($R > 0$), and green indicates negative correlations ($R < 0$). Deep color indicates correlation with statistical significance ($P < 0.05$), and light color indicates correlation without statistical significance ($P \geq 0.05$).
