# Supplementary material for: Human-derived fecal microbiota transplantation alleviates social deficits of the BTBR mouse model of autism through a potential mechanism involving vitamin B6 metabolism
Source: mSystems. 2024 May 23;9(6):e00257-24. doi: 10.1128/msystems.00257-24 (PMC11237617; doi:10.1128/msystems.00257-24)
Supplement: Fig. S7 — Network analysis of paired colon-content metagenomics and plasma metabolomics data using MetOrigin. [file msystems.00257-24-s0007.pdf]

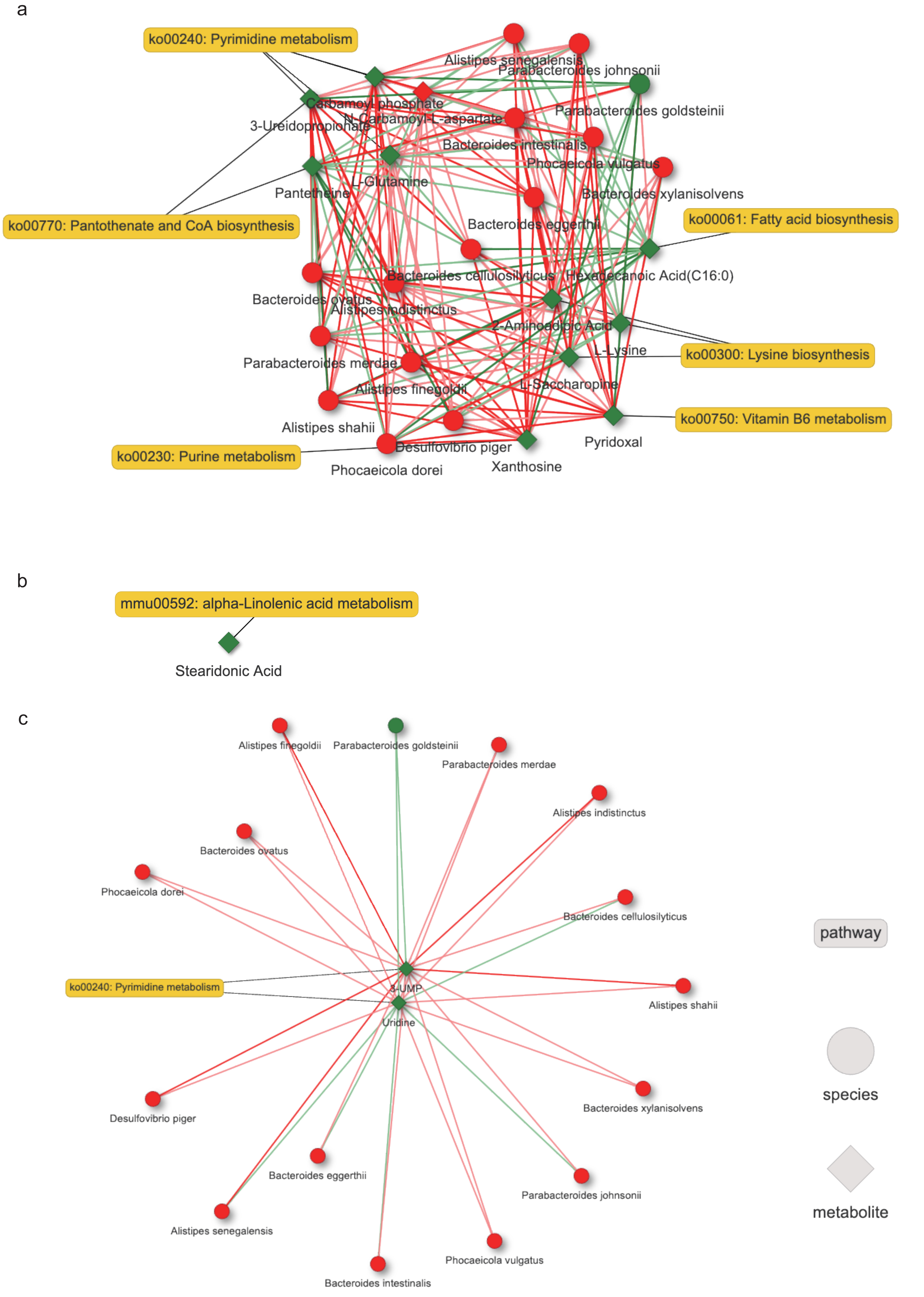
a
ko00240: Pyrimidine metabolism
Alistipes senegalensis
Parabacteroides johnsonii
Carbamoyl phosphate
N-Carbamoyl-L-aspartate
3-Ureidopropionate
Parabacteroides goldsteinii
Bacteroides intestinalis
Phocaeicola vulgatus
L-Glutamine
Pantetheine
Bacteroides xylanisolvens
ko00770: Pantothenate and CoA biosynthesis
ko00061: Fatty acid biosynthesis
Bacteroides eggerthii
Bacteroides cellulosilyticus
Hexadecanoic Acid(C16:0)
Bacteroides ovatus
Alistipes indistinctus
2-Aminoadipic Acid
L-Lysine
ko00300: Lysine biosynthesis
Parabacteroides merdae
Alistipes finegoldii
L-Saccharopine
ko00750: Vitamin B6 metabolism
Alistipes shahii
Pyridoxal
Desulfovibrio piger
ko00230: Purine metabolism
Phocaeicola dorei
Xanthosine
b
mmu00592: alpha-Linolenic acid metabolism
Stearidonic Acid
c
Alistipes finegoldii
Parabacteroides goldsteinii
Parabacteroides merdae
Alistipes indistinctus
Bacteroides ovatus
Phocaeicola dorei
Bacteroides cellulosilyticus
pathway
ko00240: Pyrimidine metabolism
3-UMP
Uridine
Alistipes shahii
species
Desulfovibrio piger
Bacteroides xylanisolvens
Bacteroides eggerthii
Parabacteroides johnsonii
metabolite
Alistipes senegalensis
Phocaeicola vulgatus
Bacteroides intestinalis

Figure S7. Network analysis of paired colon-content metagenomics and plasma metabolomics data using MetOrigin.

a Co-metabolism network connecting differential metabolites shared by the host and microbiota to the related microbes. **b** Host network shows interactions between differential metabolites with host origin. **c** Microbiota network shows interactions between differential microbial-specific metabolites and related microbes. Node shape: rectangle indicates pathway name; diamond indicates metabolite, and dot denotes microbe. Node color: red indicates upregulated metabolite or microbe, and green indicates downregulated metabolite or microbe. The node of deep color denotes statistical significance ($P < 0.05$), while the node of light color indicates no significance found ($P \geq 0.05$). Node edge: red indicates positive correlations ($R > 0$), and green indicates negative correlations ($R < 0$). Deep color indicates correlation with statistical significance ($P < 0.05$), and light color indicates correlation without statistical significance ($P \geq 0.05$).
